# Genome-wide association mapping in bread wheat subjected to independent and combined high temperature and drought stress

**DOI:** 10.1371/journal.pone.0199121

**Published:** 2018-06-27

**Authors:** Mirza Faisal Qaseem, Rahmatullah Qureshi, Quddoos H. Muqaddasi, Humaira Shaheen, Rehana Kousar, Marion S. Röder

**Affiliations:** 1 Department of Botany, PMAS- Arid Agriculture University, Rawalpindi, Pakistan; 2 Department of Breeding Research, Leibniz Institute of Plant Genetics and Crop Plant Research (IPK), Stadt Seeland OT Gatersleben, Germany; 3 Biosciences Department, COMSATS Institute of Information Technology, Islamabad, Pakistan; Institute of Genetics and Developmental Biology Chinese Academy of Sciences, CHINA

## Abstract

Most investigations to date aiming to identify the genetic basis of the stress response of wheat (*Triticum aestivum* L.) have focused on the response to single stress agents such as high temperature or drought, even though in the natural situation, these stresses tend often to occur together. Here, a panel of 108 spring type bread wheat cultivars was phenotyped for 15 yield and yield related traits for two years (2014/15 and 2015/16) under non-stressed conditions, under high temperature stress, under drought and under a combined high temperature and drought regime. The mean loss in grain yield caused by all stress regimes was 51.33%. Analysis of variance (ANOVA) of yield trait showed significant differences among genotypes environments and their interactions (G×E). All the studied traits had higher heritability values which ranged from 0.35–0.94 under [C], 0.58–0.95 under [D], 0.62–0.93 under [H] and 0.60–0.95 under [HD]. GWAS was performed by using 9,646 informative SNP markers and based on these polymorphic SNPs population structure analysis divided whole germplasm into five major sub-populations. Mixed model association analysis detected 503 marker-trait associations (MTAs) at P ≤0.001 while 329 MTAs crossed FDR ≤ 0.05 for all traits with phenotypic variances (*R*^2^) ranged from 24.83% to 12.51%. Seven new pleiotropic SNPs on chromosome 7D and IAAV8258 (86.91cM) and wsnp_Ex_c7168_12311649 (57.93cM) on chromosome 5A were most stable association in present study. Furthermore, candidate genes Psy and Sr25 (TG0040) were also significant in present study, these genes were previously mapped on 7A and 7D. The region on 7D was assiociated with 7DL.7Ag translocation from *Lophopyrum* carring rust resistance *Yr16* and many other genes. Similarly region on chromosome 7A which was associated with *Psy* gene was linked with grain yellow pigment content QTLs. Favourable alleles controlling grain yield were of vital importance and incorporation of these alleles after validation through marker assisted selection and fine mapping could be helpful in wheat yield improvement stress and non-stress conditions.

## Introduction

With respect to the volume of production, among the cereals that of wheat is second only to maize. Its productivity in the coming decades, like that of most major crops, is threatened by impending climate change: the global mean temperature is predicted to rise by 0.3°C per decade [1, 2]. As a plant adapted to temperate climates, the growth and fertility of wheat are comprised by both high temperature and moisture stress. Drought stress reduces plant height, leaf area, grain weight and grain number [3, 4]. At the physiological level, the plant responds by limiting stomatal aperture, thereby decreasing its photosynthetic rate and raising the cellular content of damaging reactive oxygen species. Unfavorably high temperatures occurring at anthesis are known to compromise pollen viability and thereby reduce grain set, while their effect post anthesis is to reduce grain size by shortening the duration of the grain filling period [5, 6]. At the physiological level, the effect of the stress is to inhibit photosynthetic electron transport [7]. Although the plant responses to drought and high temperature stress are commonly assessed in isolation from one another, in nature these two stresses often coincide. Their effect is synergistic [8, 9], altering metabolism and gene expression in ways which are different from what is induced by each stress acting independently [10]. Thus, a better understanding of their combined effect will become increasingly important if wheat improvement programs are able to keep pace with climate change.

The association mapping (AM) approach combines phenotypic with genotypic data to identify genomic locations associated with variation in a given trait. Its advantage over the more conventional bi-parental mapping approach lies mainly in the greater extent of allelic variation which can be surveyed, while also avoiding the need to establish a customized mapping population [11]. Its ability to resolve marker/trait associations (MTAs) depends upon the extent of linkage disequilibrium (LD) present in the association panel [12, 13]. An important factor influencing LD is the genetic relationship between entries, which can be readily inferred from genome-wide genotypic datasets [14, 15]. The AM approach has been applied to a range of crop species, facilitating the discovery of many quantitative trait loci. In wheat, for example, loci associated with variation in kernel size and milling quality [16], grain yield [17], resistance to disease [17–20] and various agronomic traits [21–25] have been located in this way.

Most of the AM analyses of bread wheat focusing on its stress response have to date treated drought stress separately from high temperature stress [12, 26–29], although the two stress agents are known to interact [30, 31]. The present objectives were to determine both the individual and combined effects of high temperature and drought stress on a range of yield traits in a panel of spring wheats, to characterize LD and to identify markers potentially associated with yield.

## Materials and methods

### Plant material and growing conditions

The association mapping (AM) panel comprised of 98 CIMMYT and 10 Pakistani spring wheat entries, all of which were high yielding under rain-fed conditions **(S1 Table)**. The germplasm was provided by the National Agriculture Research Center's Wheat Wide Crosses Laboratory (Islamabad, Pakistan). Six seeds of each entry were sown in three independent replications over two cropping seasons (2014/15 and 2015/16) in pots (30 cm diameter, 40 cm depth) containing sandy loam soil. After thinning three plants of each genotype were grown till maturity and after harvest two plants per pot and six plants per entry from one treatment were sampled to record yield and yield related traits. The experiments were performed in a glass house under natural daylight at National Agriculture Research Center, Islamabad (33.38°N, 73.00°E). The experiments were set out in a factorial randomized complete block design, imposing four treatments viz., control [C], high temperature [H], drought [D] and combined high temperature and drought [HD]. After anthesis the pots with [H] and [HD] treatments were shifted to the glass house where the temperature was maintained at 36±5°C. For drought treatment in [D] and [HD] the moisture content of posts was maintained at 30% of the total available water capacity by using a TDR soil moistquaure meter. To avoid permanent wilting, the pots were watered (400 ml water) when the moister declined to <20% of the total available water capacity. The stress treatments were imposed for 16 days post-anthesis following [32] after which all plants were returned to the [C] regime.

### Phenotypic data

At physiological maturity, six plants per genotype from one treatment were sampled to obtain yield-associated traits e.g., spikelet number per spike, harvest index, grain yield and number of grains per spike. Additional traits viz., number of days to heading (when 50% of the spikes emerged from the boot), days to anthesis (first appearance of exerted anthers),time taken to reach physiological maturity (when peduncle of the spike was completely yellow), plant height (measured from the base of the plant to the tip of main tiller excluding the awns), tiller number per plant, peduncle length (measured from the first node to the lowest spikelet on the leading tiller), peduncle extrusion (measured from the flag leaf ligule to the lowest spikelet on the leading tiller), spike length (excluding the awns) were also noted. Harvest index (*HI*) and leaf area (*LA*) were also measured.

### Phenotypic data analysis

The mean value for each trait was used to calculate best linear unbiased estimates (BLUEs) assuming a fixed genotypic effect, calculated using software GenStat *v*18 (VSN International, Hemel Hempstead, UK). The broad sense heritability for each trait under individual stress treatment was calculated from the expression:

$$H^{2}=\sigma_{G}^{2}/(\sigma_{G}^{2}+(\frac{\sigma_{G\times E}^{2}}{ny})+(\frac{\sigma_{e}^{2}}{ny\times nr})$$

where $\sigma_{G}^{2}$ is genotypic variance $\sigma_{G\times E}^{2}$ is variance for genotype and evironment interaction, *ny* is number of years and *nr* number of replications. The analysis of variance and estimation of inter-trait correlations were performed in the software R (http://www.r-project.org/).

### Genotyping and genotypic data analysis

DNA was extracted from seedlings using CTAB method [33]. SNP genotyping was performed by Trait Genetics GmbH (http://www.traitgenetics.com/en/), using a 15k Illumina SNP chip developed from the 90k iSelect chip described by [34] generated 13,006 SNP markers. SNPs with minor allele frequency (MAF) ≤0.05 and missing values >5% were removed from the subsequent analysis which left a set of 9,646 polymorphic SNP markers. Powermarker *v*3.25 (http://statgen.ncsu.edu/powermarker) software was used to assess the polymorphic information content (PIC) and the major allele frequencies for each SNP. Population structure was estimated from SNP markers by a Bayesian model based approach implemented in software STRUCTURE *v*2.3.3 [15] to assign the individuals into subpopulations (*K*) based on their genotype. The number of *K* from 1 to 10 was set using 100,000 burn-in iterations followed by 100,000 MCMC (Markov-Chain Monte Carlo) iterations. To estimate the sampling variance of the inferred population structure, 10 independent runs were performed for each value of *K*. The most likely number of *K* was determined by following [35] with the help of Structure Harvester software [36].

### Association mapping and linkage disequilibrium

Association analysis was performed by using each trait’s BLUE values via linear mixed-effect model following [37, 38] implemented in R package GAPIT (Genome Association and Prediction Integrated Tool) [39]. The correction for population stratification and cryptic relatedness was performed by employing coefficient of co-ancestry kinship and first three principle components as random effects in the linear mixed-effect model [40]. The significance threshold for P value was calculated based on the method of [41] (P = 1/n, n = total number of SNP used) which was equal to log10 (P) _3.98. After application of Bonferroni correction the log10 (P) threshold rose to 5.28 [42]. To check the appropriateness of the implemented model for association analysis, quantile-quantile (qq) plots were drawn between the observed and expected log_10_(*P*) values. Chromosome-wise and genome-wide linkage disequilibrium (LD) decay analysis was performed by calculating pairwise marker allele squared correlation (*r*^2^) and plotting the *r*^2^ values against the genetic distances (cM) in GenStat *v*18.

## Results

### Phenotypic variation and broad sense heritability

**Fig 1:**
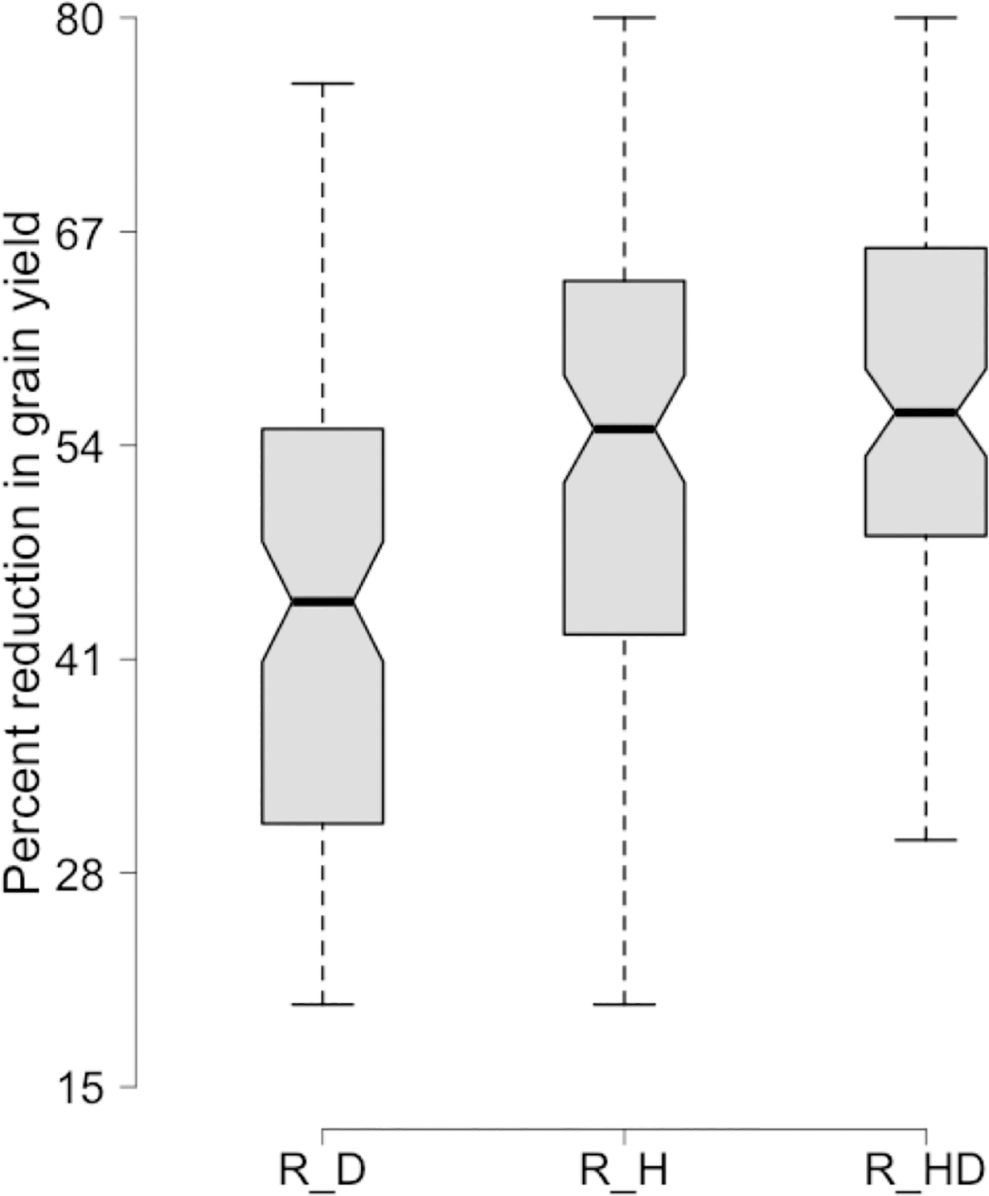
Percent reduction in grain yield under stress and non stress conditions. R_D is reduction in grain yield under drought stress R_H percent reduction in grain yield under heat stress and R_HD percent reduction in grain yield under combined drought and heat stress.

**Fig 2:**
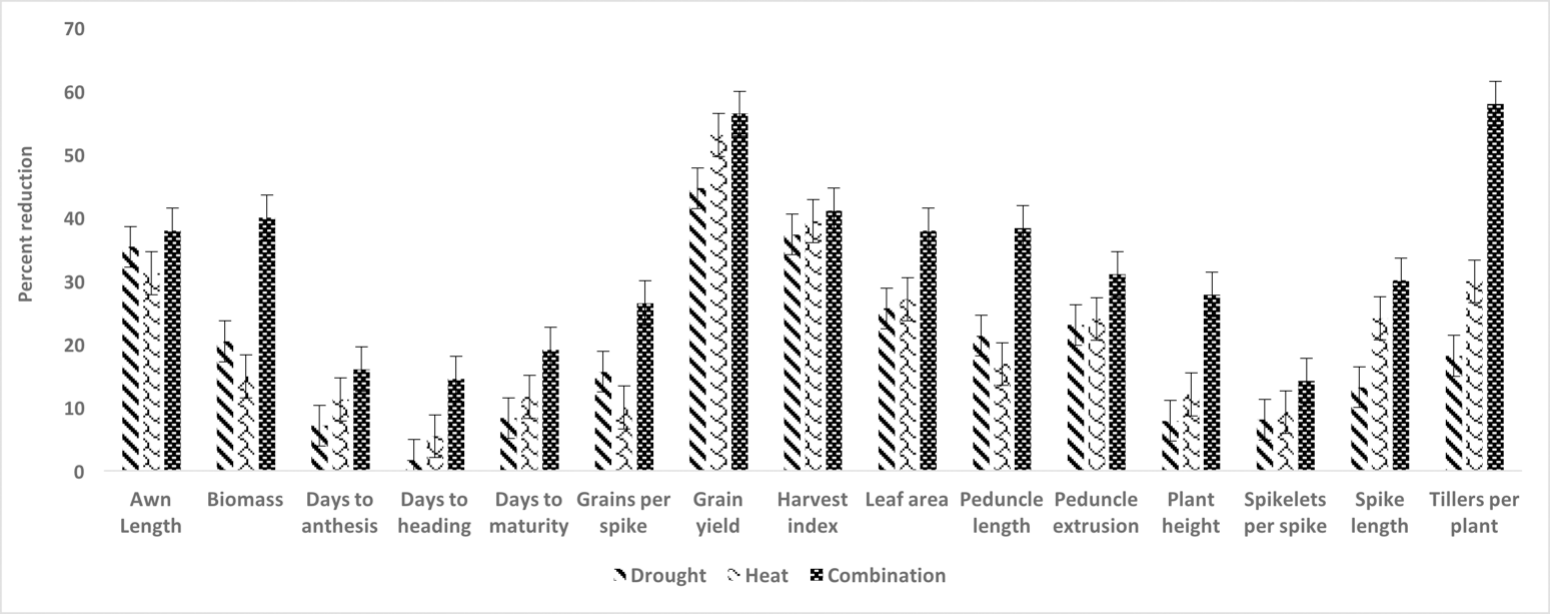
The reduction in performance induced by exposure to abiotic stress. Each column represents a treatment regime while bars at top of column represents the standard error bar.

Trait means, standard errors and coefficients of variation in response to each of the treatments are given in (**S2 Table)**. Under all the stress treatments, grain yield was normally distributed with a lower mean, compared to that achieved in the [C] treatment, in each of the stress treatments. Grain yield was most severely affected by the [HD] treatment than by either the [H] or the [D] treatment (**Fig 1**). AWL, Biomass, FLL, GPS and PDL were all more strongly compromised by drought, while the number of days to anthesis, to maturity and to heading, as well as grain yield, harvest index, leaf area, plant height, spike length, number of spikelets per spike and number of tillers per plant were more effected by the high temperature stress (**Fig 2**). HI, LA, PDL and TILL had significant and positive correlation with grain yield under three treatments i.e. [C], [D] and [HD] while DTH had positive correlation with GY under [C], [D] and [H]. AWL, GPS, Pext and PH had positive correlation with yield under [D] and [HD] while SLP had positive association with yield under [D] and [H] and Biomass and SL had positive association under [C] and [D]. The detailed correlations of grain yield with all studied traits are reported in (**S3 Table**). The broad sense heritability of the various traits under [C] ranged from 0.35 (number of days to anthesis) to 0.94 (plant height), under [H] from 0.62 (number of spikelets per spike) to 0.93 (leaf area), under [D] from 0.58 (number of spikelets per spike) to 0.95 (number of days to maturity), and under [HD] from 0.60 (number of tillers per plant) to 0.95 (leaf area) (**S2 Table, S1 Fig**). Genotype, treatment, environments (growing years) and their interactions were all significant (P<0.001) determinants of all of the yield traits (**S4 Table**).

### Linkage disequilibrium and population structure

**Fig 3:**
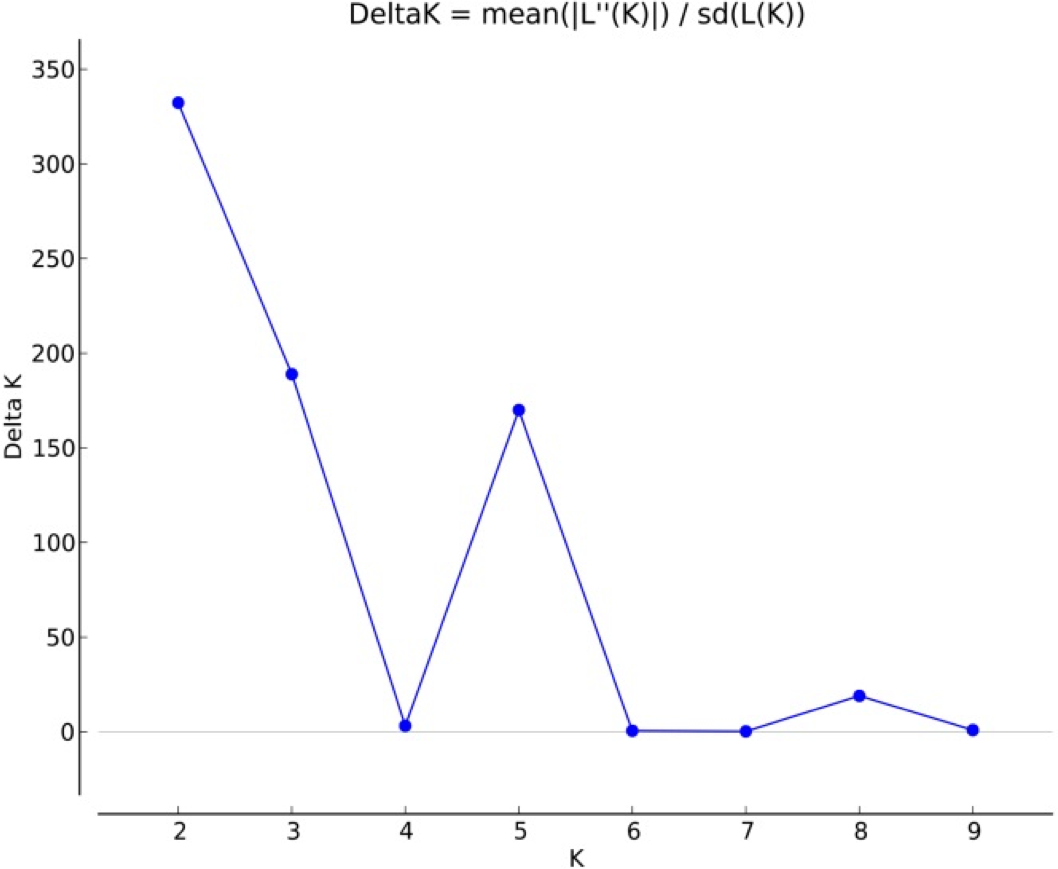
STRUCTURE analysis used to define genetic relationships in the AM panel. Δ*k* plot, with *k* ranging from 1–10.

**Fig 4:**
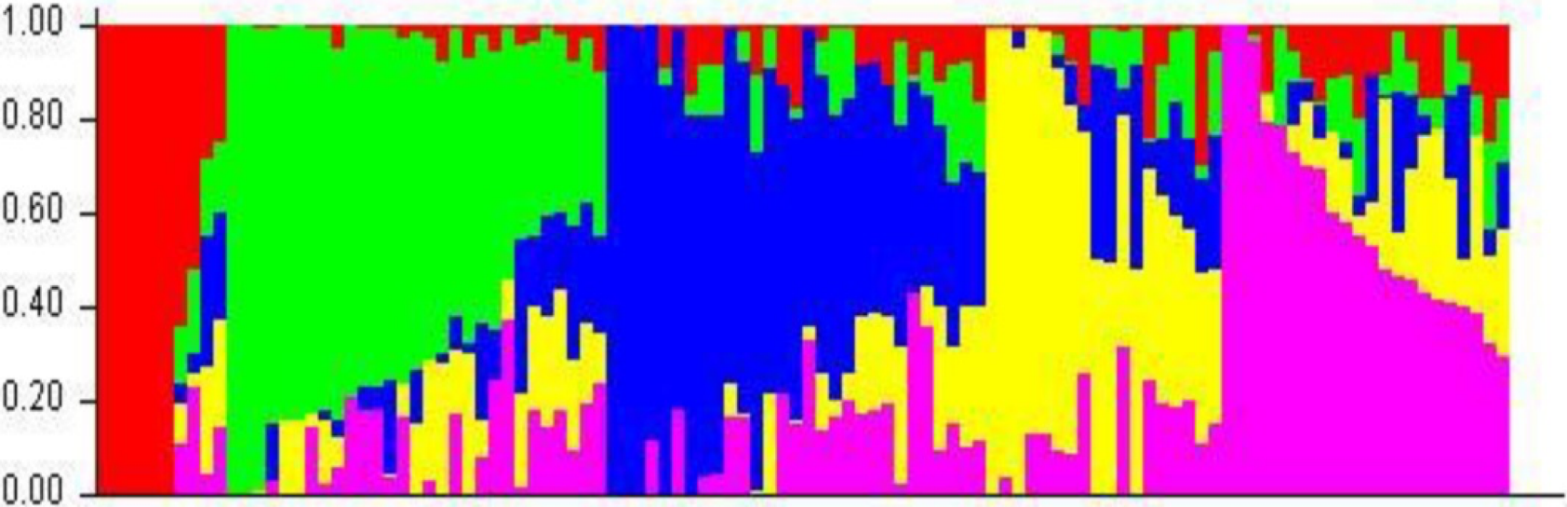
STRUCTURE analysis used to define genetic relationships in the AM panel. Each horizontal entry represents one AM panel entry. The existence of five sub-populations was inferred.

Of the set of 9,646 informative SNP markers, 4,529 defined loci on a B genome chromosome, 3,278 loci on an A genome chromosome and 1,050 on a D genome chromosome, leaving 789 markers chromosomally non-assigned (**S5 Table**). The global PIC was 0.27, varying from chromosome to chromosome from 0.35 (chromosome 6B) to 0.14 (chromosome 5D) (S4 Table). LD was greatest on chromosome 4D (r^2^ = 0.64) and lowest on chromosome 5A (r^2^ = 0.38). On the basis of the R^2^ model, the mean genetic length of these associated groups was 8.1 cM while mean Pearson’s correlation r^2^ value was 0.45 (**S6 Table**, S1 File). According to the STRUCTURE analysis, the most likely *k* value was 5 (**Fig 3**). The ten entries (9.2% of the panel) assigned to sub-population_1 included mainly of FRNCLN, MILAN and CROC_1 background; the 29 entries (26.8%) assigned to sub-population_2 had breeding lines of KAUZ, PFAU/SERI.1B, PRL/2*PASTOR and WHEAR background; the 29 entries (26.8%) assigned to sub-population_3 were of SOKOLL, VORB and PASTOR background; the 18 entries (16.6%) assigned to sub-population_4 developed from FRET2, WBLL4 and PFAU; finally the 22 entries (20.37%) assigned to sub-population_5 included entries developed from the KACHU, WAXWING and WBLL1 background **(Fig 4**). The Nei’s genetic distances between entries and a derived phylogenetic representation are shown in **S2 Fig**.

### Association mapping

**Fig 5:**
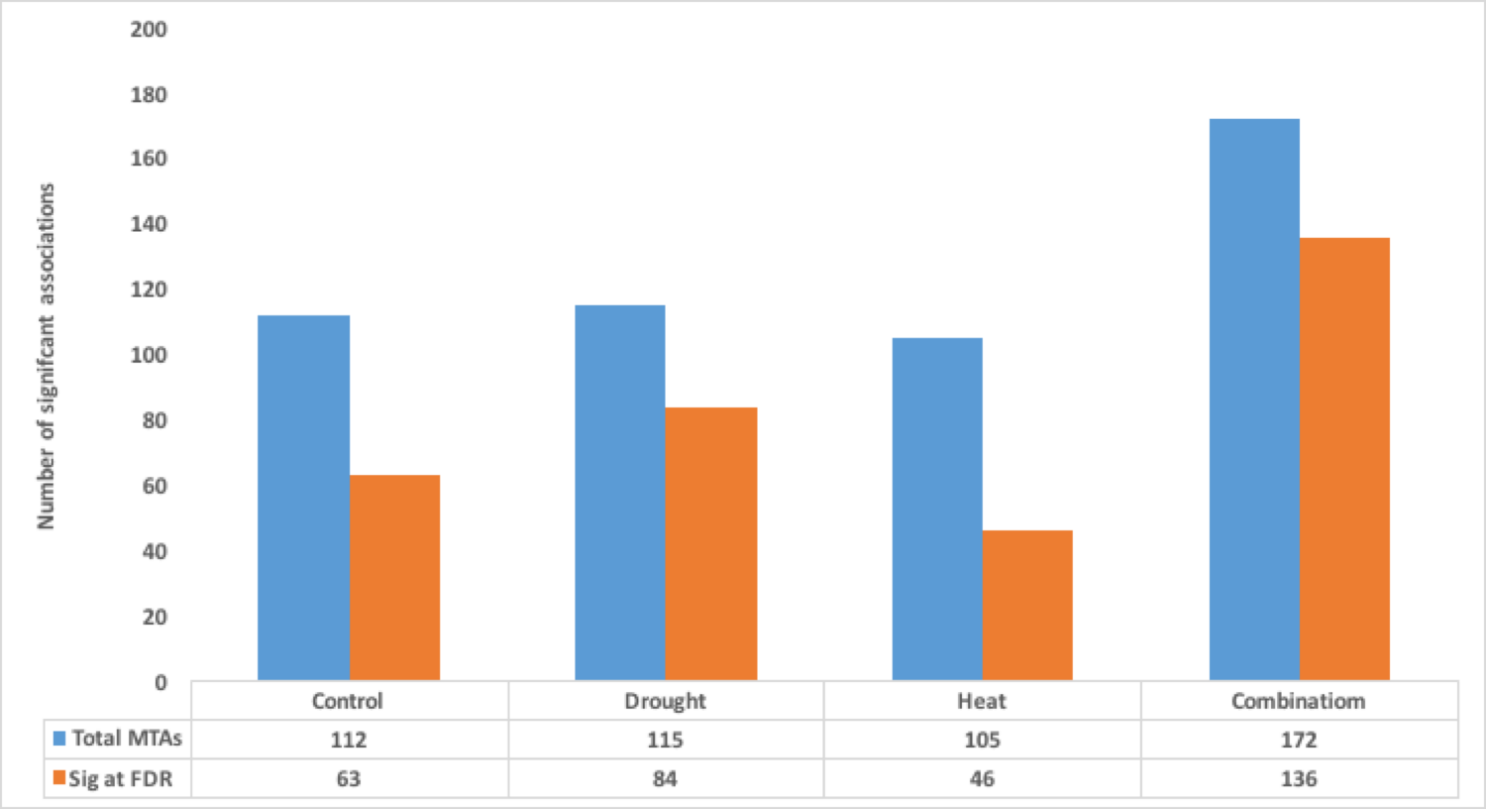
MTAs derived from the AM analysis. **The number of MTAs significant at P<0.001 and FDR < 0.05 under each stress regime.** Sig MTAs: Marker trait associations significant at P<0.001. Sig at FDR: Marker trait associations significant at FDR<0.05. C: Control, D: Drought, H: Heat HD: Combined Heat and Drought stress.

Genome wide association analysis was performed using 15k SNP markers for yield related traits in bread wheat grown in four different growing conditions viz.[C], [D], [H] and [HD]. The analysis was based on BLUE values and significance threshold was set by following Li *et al*., [41] and significance was further confirmed by using Bonferroni correction method. The present study recorded 503 MTAs significant under both stress and non-stress conditions, maximum number of associations (172) were recorded under [HD] treatment, followed by [D] with 115, [C] with 112 and [H] treatment with 104 MTAs (**Fig 5**). From these significant MTAs 63 marker trait associations crossed FDR≤0.05 threshold under [C] treatment, while 84, 46 and 136 MTAs were significant under [D], [H] and [HD] treatment, respectively (**Fig 5**). The linkage map of significant marker trait associations is shown in (S2 File).

Significant MTAs under [C] treatment explained on average 16.6% phenotypic variation with maximum contribution by leaf area explaining 21% phenotypic variation while all MTAs under [D] stress on average explained 17% variation with maximum contribution by plant height. Under [H] treatment harvest index had maximum contribution to averaged phenotypic variation and all traits on average explained 15% phenotypic variation and under [HD] treatment spikelets per spike explained maximum averaged variation and MTAs significantly associated with all traits on average explained 15% phenotypic variation **S7 Table**.

All significant MTAs under [C] had positive allelic effect on studied traits ranging from 6.36 (Peduncle extrusion) to 36.72 (Harvest index) while under [D] treatment effect size ranged from -62.72 to 52.53 and SNPs with maximum and minimum allelic effect were associated with harvest index. In [H] stress treatment allelic effect ranged from 4.35 to 59.49 with SNP significantly associated with PEXT had least allelic effect and SNP associated with harvest index had highest value of allelic effect. SNP with least allelic effect under [HD] treatment was associated with PDL (1.80) while SNP with highest effect was associated with awn length (64.80). The additive effects of markers significantly associated in all three stress treatment based on BLUEs value are shown in (**Table 1, S3 Fig)**.

**Table 1:** Loci contributing variation in yield traits and yield related traits in the AM panel plants grown under four treatment regimes: Non-stressed ([C]), high temperature ([H]), drought ([D]) and combined high temperature and drought ([H+D]).

Trait	Treatment	No of significant MTAs	MTAs significant at FDR	Percent of variation explained	Effect size
**AWL**	**Control**	2	2	19.15	18.98
	**Drought**	14	13	18.12–14.41	7.70–5.89
	**Heat**	7	6	15.83–13.05	12.39–7.49
	**Combination**	19	19	18.9–12.91	2.81–1.80
**Biomass**	**Control**	3	0	16.2	13.53
	**Drought**	4	0	17.29–16.18	20.39–19.72
	**Heat**	5	0	15.90–15.09	19.05–13.66
	**Combination**	14	13	16.57–11.26	23.71–12.68
**DTA**	**Control**	4	0	16.10–15.71	14–13.30
	**Drought**	10	10	25.67–15.02	12.23–9.41
	**Heat**	4	0	16.97–15.30	16.67–15.49
	**Combination**	7	3	17.31–13.03	15.01–10.86
**DTH**	**Control**	1	0	16.17	12.14
	**Drought**	4	2	20.04–16.04	6.76–5.35
	**Heat**	9	8	23.39–16.60	31.12–16.46
	**Combination**	7	0	15.33–12.80	12.08–9.63
**DTM**	**Control**	1	0	15.72	17.22
	**Drought**	11	3	201.19–15.31	17.11–5.45
	**Heat**	1	0	15.24	14.56
	**Combination**	10	10	14.94–11.36	22.59–15.53
**GPS**	**Control**	12	12	21.03–16.14	14.48–10.44
	**Drought**	5	2	21.59–17.26	4.61–4.81
	**Heat**	9	0	15.85–15.68	26.44–26.30
	**Combination**	11	8	18.77–13.04	35.97–22.36
**GY**	**Control**	9	0	20.95–15.44	12.7–8.31
	**Drought**	11	11	20.58–12.41	29.71–16.75
	**Heat**	12	8	21.17–15.68	8.91–6.90
	**Combination**	13	8	23.46–12.37	9.70–4.92
**HI**	**Control**	16	13	20.36–15.28	36.72–26.35
	**Drought**	11	10	20.44–14.75	52.53–62.72
	**Heat**	1	0	19.64	59.49
	**Combination**	4	1	27.99–14.35	20.83–4.73
**LA**	**Control**	3	3	21.30	22.25
	**Drought**	11	6	18.31–14.12	41.24–30.69
	**Heat**	4	0	20.81–15.59	27.27–22.56
	**Combination**	7	7	20.98–17.05	13.35–12.60
**PEXT**	**Control**	3	0	15.60–15.37	6.36–8.03
	**Drought**	10	10	24.83–16.41	16.71–11.46
	**Heat**	16	16	21.21–16.05	7.12–4.34
	**Combination**	14	10	21.04–12.57	8.22–4.48
**PH**	**Control**	10	8	18.64–17.31	25.77–20.50
	**Drought**	3	2	22.82–16.41	5.46–4.06
	**Heat**	6	0	14.99	15.18
	**Combination**	9	9	21.68–12.95	32.03–19.49
**PL**	**Control**	13	0	17.82–14.33	17.30–13.33
	**Drought**	1	0	16.50	11.14
	**Heat**	10	0	17.71–15.45	16.20–13.57
	**Combination**	12	11	18.36–9.95	64.80–35.43
**SL**	**Control**	10	8	19.54–14.51	19.42–13.17
	**Drought**	8	8	14.39–14.22	9.78–8.08
	**Heat**	3	0	16.52	11
	**Combination**	11	10	14.12–9.45	13.95–8.15
**SLP**	**Control**	11	8	21.37–14.89	20.61–12.41
	**Drought**	1	0	12.27	7.58
	**Heat**	NA	0	NA	NA
	**Combination**	1	0	20.40	12.68
**TILL**	**Control**	14	9	19.02–13.98	36.36–21.32
	**Drought**	5	1	20.74–16.57	10.79–8.70
	**Heat**	12	6	20.62–15.76	10.66–7.39
	**Combination**	12	6	20.62–15.76	11.32–6.7
**Percent reduction in GY**	**Drought**	6	6	19.38–17.41	0.44–0.36
	**Heat**	5	2	20.63–15.66	0.30–0.26
	**Combination**	21	21	16.88–13.29	0.64–0.48

AWL: Awn length, Biomass: Above ground plant dry weight, DTA: Days to anthesis, DTH: Days to heading, DTM: Days to maturity, GPS: Grains per spike, GY: Grain yield, HI: Harvest index, LA: Leaf area, PL: Peduncle length, Pext: Peduncle extrusion, PH: Plant height, SLP: Spikelets per spike, SL: Spike length, Till: Tillers per plant

### Potent markers controlling grain yield

**Fig 6:**
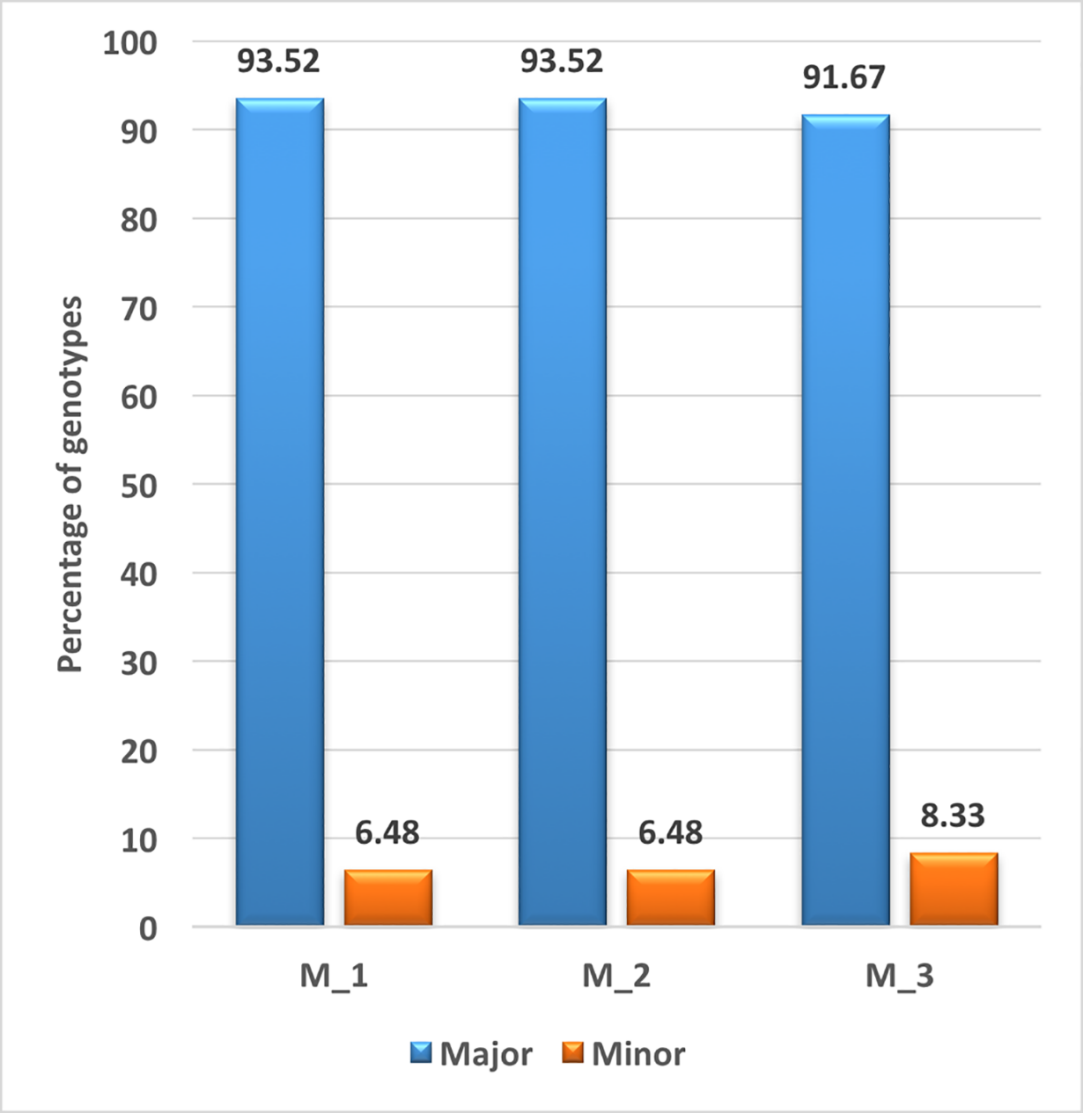
Percentage of genotypes containing major and minor alleles under all stress treatment M_1 and M_2 were same markers significantly associated under [HD] and [H] treatments while M_3 was associated with GY under [D] treatment.

**Fig 7:**
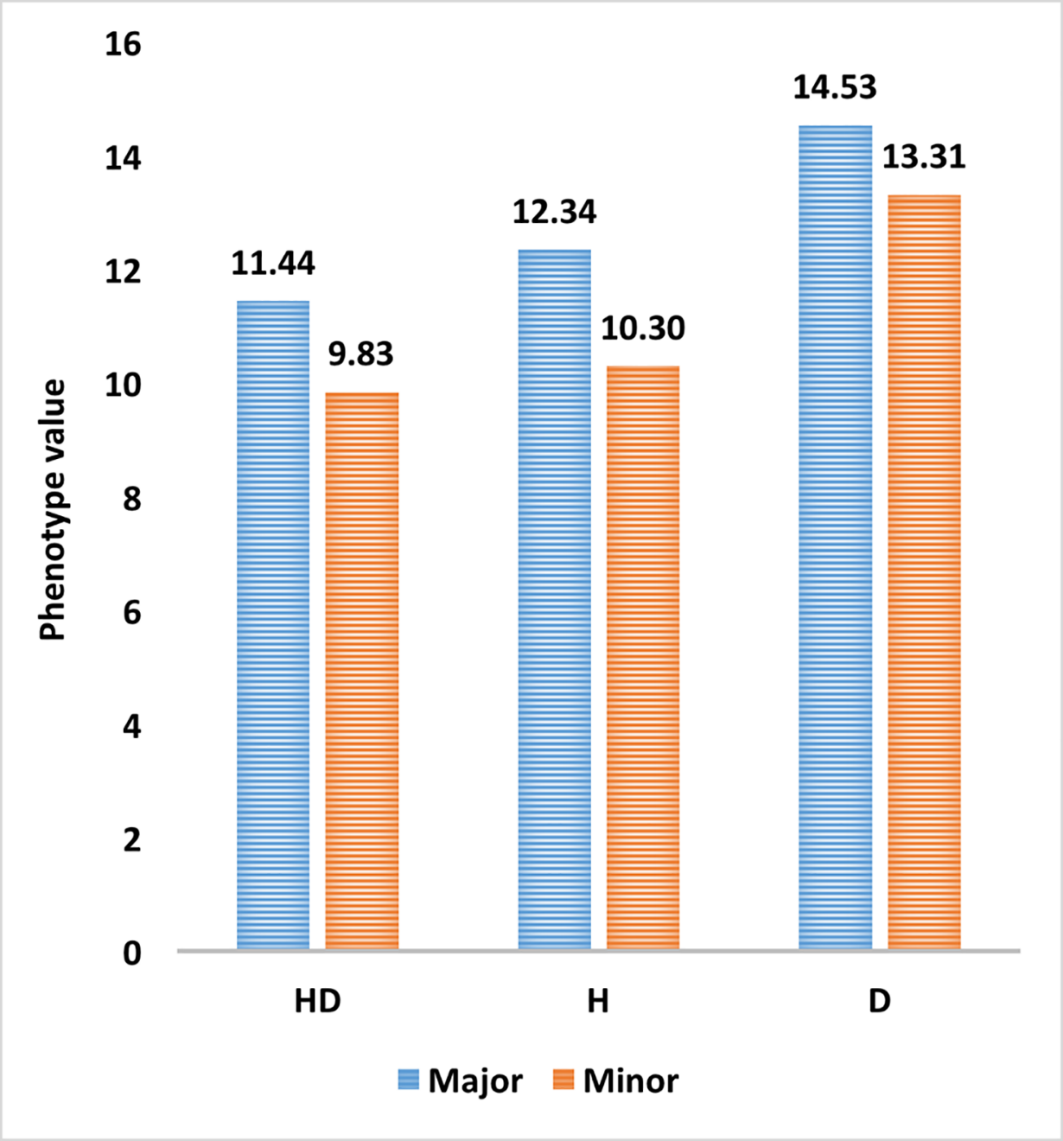
Phenotypic value of grain yield for genotypes carrying favorable (Major) allele in different stress treatments. Major: favorable allele Minor: Unfavorable allele, H: Heat, D, Drought and HD: Combined heat and drought stress.

Marker *wsnp_BM134363A_Ta_2_4* on unmapped chromosome was associated with grain yield under two out of four treatments while marker RAC875_rep_c69171_92 on chromosome 3B was associated was associated with grain yield under [H] treatment. The first marker had allelic effect of 29.71 and later had effect size of 9.70. In all the three treatments mainly major alleles are more favorable in enhancing grain yield. Out of total genotypes 93.5% genotypes carried favorable allele of marker *wsnp_BM134363A_Ta_2_4* (M1) and only 6.48% genotypes carried other allele **Fig 6**. Similarly, 91.67% genotypes carried favorable allele of marker RAC875_rep_c69171_92 while 8.33% of total genotypes had other allele. The favourable allele caused 16.32, 19.90 and 9.20% increase in yield under [HD], [H] and [D] treatments respectively **Fig 7.**

The detailed summary of GWAS results, number of MTAs significant at FDR≤0.05, phenotypic variation and allelic effect size of each significant MTAs for each trait is given in **Table 1, S7 Table.**

### Stress specific MTAs

**Fig 8:**
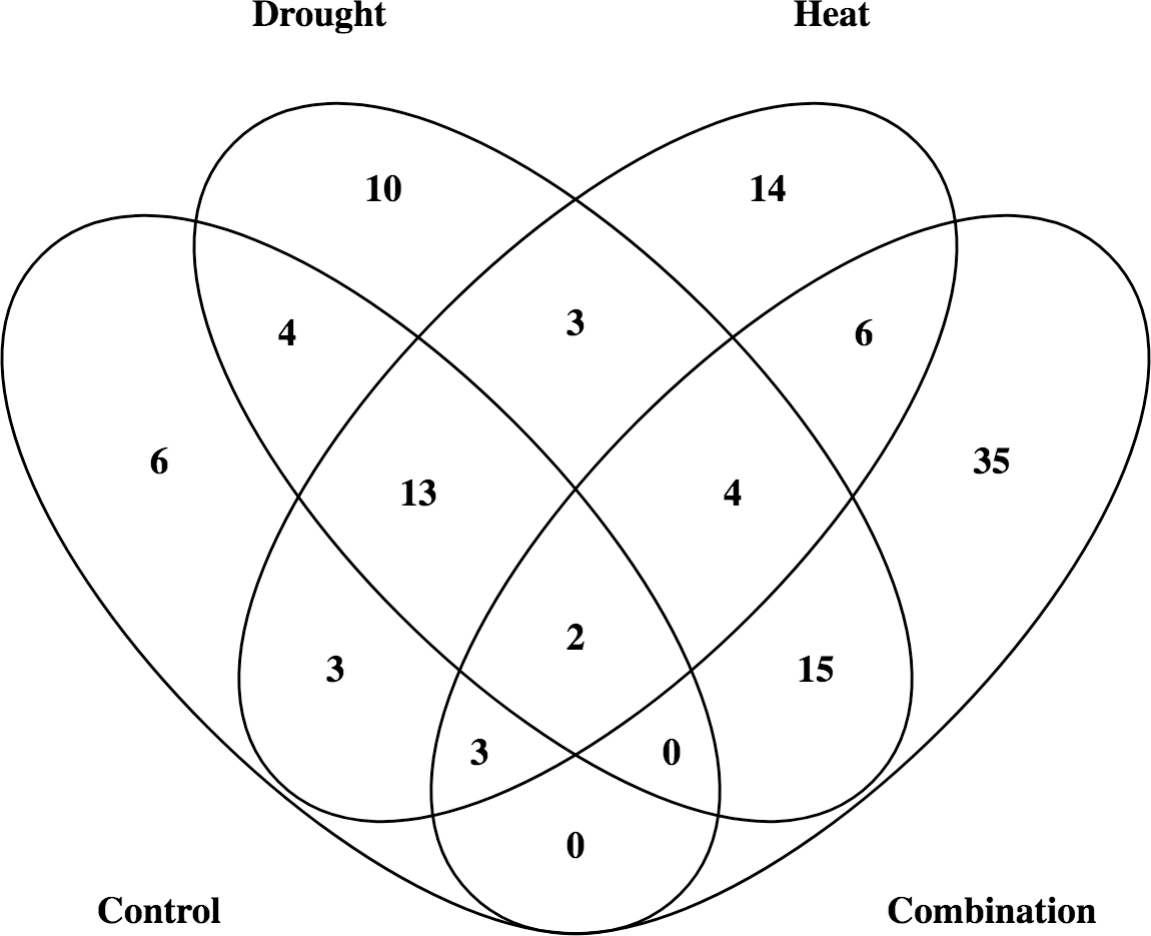
Shared and unique marker trait associations among different stress treatments. Number represent number of associations common between two or more than two treatments.

Very few stress specific MTAs were recorded in the present study, maximum number of stress specific MTAs were recorded under [HD] treatment followed by [H], [D] and [C] treatments each having 35, 14, 10 and 6 associations respectively. Maximum number of associations (15) was shared among [D] and [HD] treatments while no association was shared among [C], [D] and [HD]. Similarly, [C] and [HD] treatments also have no shared associations (**Fig 8**). IAAV8258 (86.91cM) and wsnp_Ex_c7168_12311649 (57.93cM) present on chromosome 5A were two stable associations common among all treatments. The detailed information about stress specific and MTAs common between two or more than two stresses is given in **(Fig 8)**.

### Pleiotropic MTAs

A total 24, 24, 28 and 19 pleiotropic MTAs were recorded under [C], [D], [H] and [HD] treatment, respectively. Pleiotropic associations under [C] treatment were concentrated on chromosome 5A and markers *wsnp_Ex_c21633_30782312* and *BS00093036_51* located on chromosome 6A at 6.98 cM were associated with maximum (7) traits. Under [D] treatment, most of pleiotropic MTAs were present on unmapped chromosome followed by 7A between 137.39–161.31cM, marker *wsnp_JD_c2578_3489735* and *BS00077768_51* on chromosome 2A were associated with five traits. Under [H] treatment chromosome 3B contained maximum number of pleiotropic MTAs between 8.95–62.57cM. Most of pleiotropic regions concentrated on chromosome 7A were significant under [HD] treatment, marker *wsnp_CAP7_c1321_664478* and *wsnp_CAP7_c1321_664480* on chromosome 7A were associated with 12 traits. Furthermore, these pleiotropic regions were divided into 7 different clusters, detailed information about these clusters and associated traits is presented in **Table 2**.

**Table 2:** Summary of pleiotropic QTL detected in the spring wheat association mapping population exposed to independent and combined heat and drought stress.

Locus	Chromsome	Position	Traits			
			Control	Drought	Heat	Combination
wsnp_JD_c2578_3489735~ Excalibur_c52319_257	2A	134.75~149.83	SL, SLP, TILL	DTA, DTH, GY, PEXT, PH	AWL, DTH	AWL, BIOMASS, DTA, DTM, GPS, GY, PL, SL
wsnp_Ra_rep_c74497_72390803~Jagger_c7688_98	2B	119.07~134.46	DTH	SL	BIOMASS, PEXT, GY, TILL	TILL
RAC875_rep_c69171_92~Ra_c58335_189	3B	8.95~12.65	GPS, GY, HI		DTA, GY, TILL	TILL
BobWhite_c12908_381~wsnp_BE498786B_Ta_2_1	3B	34.61~62.57		BIOMASS	GY,PEXT,PL,TILL	TILL
BobWhite_c2236_111~wsnp_Ex_c7168_12311649	5A	30~57.93	Biomass, GPS, GY, HI, LA, PL	Percent reduction in GY	GPS, PSL, SL	BIOMASS, DTH, DTM, GPS, GY, LA, PEXT, PH, PL, SL, SLPS,Percenmt reduction in GY
wsnp_CAP7_c1321_664478~IACX7848	7A	148.43~161.31		DTA, DTM, GY, PEXT		AWL, BIOMASS, DTA, DTH, DTM, GPS, GY, LA, PEXT, PH, PL, SL
D_F5XZDLF02FKJFM_220~Kukri_c45628_892	7D	197.58~206.75	HI, PH, PL, SL, SLP, TILL	AWL, HI, LA, SL	AWL, DTH, GPS, PEXT, PH	

AWL: Awn length, Biomass: Above ground plant dry weight, DTA: Days to anthesis, DTH: Days to heading, DTM: Days to maturity, GPS: Grains per spike, GY: Grain yield, HI: Harvest index, LA: Leaf area, PL: Peduncle length, Pext: Peduncle extrusion, PH: Plant height, SLP: Spikelets per spike, SL: Spike length, Till: Tillers per plant

Chr: Chromosome Pos: Position in Centi Morgan

### Candidate genes

In the present study candidate gene TG0040 was associated with multiple traits under both stress and non-stress conditions, this marker was part of 15K Illumina chip which is a modified version of 90K chip. TG0040 marker was associated with Psy/Sr25 genes **(S7 Table)**, QTLs for GYPC which was linked with Phytoene synthase 1 (Psy) were mapped on distal part of chromosome 7A and 7B [43, 44]. A QTL region on chromosome 7BL for GYPC linked to Phytoene synthase 1 (PSY-B1) gene suggest that is good candidate gene for this region [44]. The Sr25 on chromosome 7D was associated with Lr19 which is involved in rust resistance in bread wheat. The germplasm used in the present study contain “Seri” as one of their parent which is developed by transferring Lophopyrum 7DL.7Ag from “Oasis” to “Seri”. The translocation is present on long arm of chromosome 7D and contain many genes like *Lr19* and endosperm yellow pigment gene Y [45]and the segregation distortion factor *Sd1* [46]. The pleiotropic effect of these genes might be reason for higher yield of genotypes with 7DL.7Ag translocation under irrigated environment. 7DL.7Ag translocation caused significant increase in grain number and also improves sink strength which lead to more biomass at maturity [47, 48]. The increase in grain number and biomass in Seri genotypes was reported by [49]. In spite of having positive effects on grain yield under irrigated conditions 7DL.7Ag translocation had negative effect under drought stress which could be related to over production of biomass at early growth stages. This result in increased competition for water in the later growth stages leading to lower spike fertility and number of fertile tillers at maturity [48, 49]. Under hot environment genotypes carrying Seri parent had improved physiological traits like chlorophyll content which is cause of higher yield than genotypes with non-Seri parents [50, 51].

### Trait specific MTAs

#### Awn length (cm)

Trait specific MTAs for AWL were recorded under [D] and [HD] treatments, marker RAC875_rep_c111788 _130 (197.58cM), Kukri_c45628_892 (206.75cM) and BS00022875_51 (203.58cM) present on chromosome 7D were specifically associated under [D] while eight AWL specific MTAs were detected on chromosome 3A between 101 to 105.67cM under [HD] treatment. Significant associations under [C] treatment had higher effect size (18.98) and explained 19% phenotypic variation (S7 Table, S3 File).

#### Biomass (g)

All the markers significantly associated with Biomass under [D] stress were trait specific of which RAC875_c15713_943, BS00109052_51 at 49.73cM and BobWhite_c2236_111 at 49.61cM were present on 5A and RAC875_c3138_224 at 62.57cM on 3B chromosome. Under [H] treatment TA001218-0519, wsnp_BE489326B_Ta_2_2 and BobWhite_c16847_343 were present on chromosome 3B at 65.72cM and were specific to Biomass and under [HD] treatment BS00071183_51 on 3B at 144.74cM and Tdurum_contig56175_791 on 7A at 135.81cM were specific to Biomass(S7 Table, S3 File).

#### Days to heading

Under [C] treatment DTH had one trait specific SNP (wsnp_Ra_rep_c74497_72390803) on chromosome 2B at 119.07cM while IAAV7168 and IAAV6119 present on unmapped chromosome were specific DTH under [D] and [H] stress, respectively (S7 Table, S3 File).

#### Days to maturity

Non stress treatment [C] specific SNP (wsnp_Ex_c28204_37349164) for DTM was detected on chromosome 2A at 108.46cM. Under [D] stress treatment DTM has four stress specific MTAs, CAP8_c1066_309 and Kukri_rep_c72329_163 were present on 5A between 55-65cM and TA004548-0753 and GENE-3791_463 were present on unmapped chromosome (S7 Table, S3 File).

#### Grain per spike

All significant associations recorded for GPS under [D] stress were specific with marker Excalibur_c18172_2105 of 3B being most potent among all (S7 Table, S3 File).

#### Grain yield (g)

The only trait specific association (BS00039936_51 at 6.04cM) on chromosome 4B was detected for GY under [H] (S7 Table, S3 File).

#### Harvest index

Harvest index had IAAV6119 (unmapped chromosome) and Kukri_c8913_655 present on chromosome 3D at 119.41cM specifically associated under [D] treatment. BS00065292_51 (79.92cM) and IAAV2328 (80.63cM) at chromosome 5A and BS00066295_51 on chromosome 7A at 148.43cM were associated only with HI under [HD] treatment.

#### Leaf area

All the significant markers associated with LA under [H] treatment were trait specific and were concentrated on chromosome 1B (154.84cM), 5A (62.72cM) and 6A (6.98cM) under heat stress.

#### Peduncle extrusion (cm)

SNPs Ku_c6050_678 and Ra_c9190_1384 on chromosome 2B at 102.28cM were specifically associated with PEXT under [C] treatment. Kukri_c45628_892 (7D at 206.75cM), BS00022875_51 (7D at 203.58cM), GENE-1587_448 (3B at 62.67cM) and RAC875_rep_c111788_130 (7D at 197.58cM) were associated with PEXT under [H]. Markers BS00033442_51 (126.4cM), BS00084370_51 (126.8cM) and wsnp_CAP11_c651_429138 (128.37cM) were present on 7A and BS00066319_51 on 3A at 103.83cM were associated with PEXT under [HD] treatment (S7 Table, S3 File).

#### Peduncle length (cm)

Marker wsnp_BE405849A_Ta_1_1 on unmapped chromosome and wsnp_Ex_c7168_12311649 on 5A at 57.93cM were associated with PDL under [H] stress treatment.

#### Spike length (cm)

Marker GENE-2220_165 on 7D at 161.13cM was specific to SL under non stress conditions while Jagger_c7688_98 and BS00011047_51 on 2B at134.46cM were specifically associated with SL under [D] treatment (S7 Table, S3 File).

#### Tillers per plant

Kukri_c45628_892 and BS00022875_51 on chromosome 7D at 206.75cM and 203.58cM were associated with TILL under [C] treatment. Two markers wsnp_JD_c514_781859 and Kukri_rep_c69422_54 present on unmapped chromosome were significantly and specifically associated with TILL under [D] stress. All the markers significant for TILL under [HD] were specific to TILL and these were present on chromosome 2B, 3B, 3D, 4B, 5A and on unmapped chromosome.

#### Percent reduction in grain yield

A total 6, 5 and 21 MTAs were significant associations were recorded under drought heat and combine drought and heat stress and on average they explained 18, 18 and 14% phenotypic variation with average effect size of 40, 29 nd 49 respectively. Associations for percent reduction in grain yield under drought stress were detected on chromosome 1A, 3B, 4B and 5A while under heat stress on 2B, 5A and 5D and under combined heat and drought stress on chromsoem 1A, 3B, 4B, 5A, 5B, 6A and 7A. Under [HD] 15 stress specific associations were detected and 3 associations were specific to percent reduction under [H] stress while no [D] specific association was detected (S7 Table, S3 File).

## Discussion

GWAS for yield and yield related traits was conducted for an association mapping panel consisting of 108 bread wheat accessions belonging to CIMMYT heat and drought nurseries. The germplasm showed great diversity in morphological characters and agro-traits. Many association studies reported effects of individual heat or drought on bread wheat but a very few studies are available which elucidate the combined effects of heat and drought on bread wheat. To best of over knowledge, the combination of both stresses on bread wheat was studied only by [31] and [22]. In present study, [HD] treatment had hypo-additive effect over yield i.e. the effect of [HD] treatment was higher than the individual effects of [H] and [D] treatment but was lower than their sum. The reduction in yield under [HD] treatment was due to effect of interaction of heat and drought on stomatal movements, plant have to close their stomata under high temperature in [HD] treatment which causes osmotic imbalance [52, 53]. These results are in agreement with [9, 52, 54]. The means of all the morphological traits differ considerably across the years and within the treatments, suggesting that treatments and years had more significant effect on traits than genotypes.

Among all traits DTH, HI, LA, PDL and TILL had consistent correlation with grain yield, similarly AWL, Biomass, GPS, Pext, PH, SL and SLP had positive association with yield in two treatments. Negative association between grain yield and days to maturity suggests that genotypes taking more time to mature under stress had low yield. So earliness is associated with better performance under stress and this was in agreement with [29].

Population structure is linkage disequilibrium caused by admixture of different sub-population and cause false positive associations if not taken in consideration [38, 55]. GWAS has become more complicated due to complex stratification and complex breeding history of germplasm along with limited gene flow between different subpopulations, this can be reduced by taking population structure as covariant in GWAS studies [56]. AM panel used in the present study, number of genotypes shared two or more parents and thus some clusters of genotypes were dominated by the lines which have common parentage. This sharing of parents is because few elite superior lines are consistently used as parents of crosses designed in different breeding programs and thus lead to some sort of population structure as observed in the present study.

Association mapping is based on linkage disequilibrium which is non-random association of alleles and can be effected by population type, chromosome region and mating system. Extent of LD depends upon genetic distance and it determines the number of markers required for association mapping [57]. In the present study LD decay rate was determined on chromosome level, difference in LD decay among chromosomes is useful in identifying the regions subjected to genomic selection in previous studies [58]. In present study, highest level of LD, least number of markers and slow LD decay was observed on D genome. LD decayed within ~8cM for A genome and ~5cM for B genomes, while it extended up to ~10cM genetic distance for the D genome (**S5 Table**). More LD in A and B genome is due to recent evolution of D genome about 1–2 million years compared to A and B genome which evolved about 7 million years ago [59].

Overall, 503 MTAs were identified significant at P≤0.001 in both stress and non-stress treatments out of which 329 MTAs crossed Bonferroni threshold i.e. FDR≤0.05. Although grain yield QTL were detected on all wheat chromosomes in previous studies, the present study reports significant MTAs for grain yield on chromosome 2B, 3B, 5A and 7A. The MTA results from the present study were compared with previous studies using chromosome position of significant MTAs (**S8 Table**). BS00011047_51 and Jagger_c7688_98 at 134.46cM on chromosome 2B were associated with GY under [H] treatment and were near to QTL *Q*.*Ndvi*.*aww* (131cM) associated with NDVI under heat stress in a population of 368 DH wheat lines derived from a cross between RAC875 and Kukri using DArT and SSR markers reported by [60]. A similar region about 5cM apart from BS00011047_51 and Jagger_c7688_98 was reported in another study by [61] in same population grown in six experiments over three field seasons and region was found significantly associated with TKW. Similarly, BobWhite_c12908_381 on 3B was associated with grain yield [H] and tillers per plant was 5cM apart from significant region associated with grain filling duration reported by [29] in a panel of 287 wheat association mapping panel II develop from CIMMYT heat and drought adapted nurseries using DArT markers. Another region at 51.07cM was associated with GY under heat stress in present study which was previously reported to be associated with SPAD at grain filling in wheat association mapping initiative (WAMI) population of 287 elite, spring wheat lines using 90K SNP Illumina chip by [62].

A chromosomal region (BS00110075_51 and wsnp_Ra_c17216_26044790 at 76.81cM) on 5A was associated with GY under non stress treatment in the present study, while a region ~10cM apart from this region was associated with thousand grain weight in a population of 105 elite wheat varieties by [63]. Genomic region (BS00071424_51) on chromosome 7A at 137.39cM was associated with grain yield under [D] and [HD] treatment, a nearby region (126.80cM) to this consistent region was associated with grain yield in a study conducted on population of population of 127 RILs [64]. Another study by [63] reported genomic regions at 130.27cM was associated with Kernel per spike.

### Co-localization of QTL for yield and related traits

Most of the member of present AM panel carrier member from Seri/Babax population thus in such populations multiple genomic regions are involved in controlling any specific trait especially under stressed environment. This reflects the complex nature of genomic response to adverse conditions. The identification of MTAs which were informative in more than one of the stress treatments suggests a level of shared genetic control of the high temperature and drought stress response. Yield under stress is mainly controlled by the QTLs controlling heading and maturity time because the duration of the crop life cycle affects the timing and intensity of the stress experienced by the plants [65, 66]. There are major genetic factors governing heading time in wheat, i.e. vernalization (Vrn) and photoperiod (Php) responsive genes and ‘earliness per se’ (Eps) [67]. In present study, seven clusters of QTLs were detected on chromosome 2A, 2B, 3B (2), 5A, 7A and 7D each associated with more than two traits and QTL cluster for GY were detected on chromosome 2A, 2B, 3B, 5A and 7A (**Table 2**).

Cluster on chromosome 2A at 134.75–149.83cM was associated with SL, SLP, TILL under [C] treatment, DTA, DTH, GY, PEXT, PH under [D] treatment, AWL, DTH under [H] treatment and with AWL, BIOMASS, DTA, DTM, GPS, GY, PL, SL under [HD] treatment. This region was the same as detected by [68] which was present at 139.6∼145.9cM and was associated with kernel number per spike (KNS), Chlorophyll content at anthesis (Chl-A), chlorophyll content ten day after anthesis (Chl-10) detected in a RILs population of 246 genotypes developed from cross between Zhou 8425B and Chinese Spring.

Cluster on 2B (119.07–134.46) was associated with DTH under [C], SL under [D], BIOMASS, PEXT, GY, TILL under [H] and TILL under [HD] treatment. A region closer to this was reported by [69] (IWA3741 at 137.9cM, IWA1765 at 148.0cM) and was associated with leaf rust response in collection of 496 durum wheat accessions. Various previous studies report *PPD-B1*gene on chromosome 2B that is involved in controlling flowering time in wheat and might be the cause for pleiotropic effect in present study (**S8 Table**).

On chromosome 3B two clusters were identified first cluster at 8.95–12.65cM was associated with GPS, GY, HI under [C], DTA, GY, TILL under [H] and with TILL under [HD]. The second cluster was between 34.61–62.57cM and associated traits included BIOMASS under [C], GY, PEXT, PL, TILL under [H] and TILL under [HD] treatment. The pleiotropic region on chromosome 3B (12.9–13.9cM) was associated with leaf green area (GA), grain filling duration (GFD), test weight (TW), thousand-kernel weight (TKW), days to heading (DH) and kernel number per spike (KNS) in study by [29] in a panel of 287 wheat association mapping panel II developed from CIMMYT heat and drought adapted nurseries using DArT markers (**S8 Table**).

The cluster on chromosome 5A between 49.61–57.93cM was associated with Biomass, GPS, GY, HI, LA, PL under [C], GPS, SLP, SL under [H] and BIOMASS, DTH, DTM, GPS, GY, LA, PEXT, PH, PL, SL, SLP under [HD] treatment this region was of prime importance as it was associated with GY under two treatments i.e. [C] and [HD], while this region was associated with GPS in three treatments i.e. [C], [H] and [HD]. Genomic region between 50.6–70.6 cM on chromosome 5A associated with plant height, spike length and internode length in a population of 188 RILs derived from Kenong 9204 × Jing 411 cross was reported by [70]. Major genes controlling spike architecture traits viz. Q, C, and S are reported on chromosome 5A of bread wheat [71]. These genes control various spike attributes like spike length, shape, as well as it has pleiotropic effects on glume tenacity, rachis fragility, plant height and spike emergence time [72, 73].

Pleotropic region on chromosome 7A between 148.43–161.31cM was associated with DTA, DTM, GY and PEXT under [D] treatment while similar region was associated with AWL, BIOMASS, DTA, DTH, DTM, GPS, GY, LA, PEXT, PH, PL and SL under [HD] treatment. This region was close to pleiotropic region (168.0–178.4cM) reported by [74] associated with TKW (thousand kernel weight), SN (spike number/m^2^), PH (Plant height), SL (spike length), and Chl-10 (SPAD value of chlorophyll content at 10 days post-anthesis) 246 RILs population derived from the cross of Zhou8425B/Chinese Spring wheat. The pleiotropy/linkage of loci contributing both to grain yield and phenology confirms that in stress-prone environment, early maturity is beneficial for grain filling, as it leads to an increase in grain size and yield (**S8 Table**).

The cluster on 7D at 197.58–206.75 was associated with HI, PH, PL, SL, SLP, TILL under [C], AWL, HI, LA, SL under [D] and with AWL, DTH, GPS, PEXT, PH under [H] treatment no such pleiotropic region was reported in literature. As the population used in present study is mainly derived from SeriM82 and Babax parents, they contain the photoperiod insensitive alleles at Ppd-D1, as well as spring-type alleles for at least two vernalization loci [22, 31]. Seri parent also contained 7DL.7Ag translocation which is know to improve yield under irrigated environments These favorable alleles of potent SNPs have potential to increase yield under both stress and non-stressed environments so these deserve to be incorporated in wheat breeding program. MTAs significant for percent reduction in grain yield may be important to understand wheat response to environmental stresses, and provides valuable clues for selection in breeding practice.

## Conclusion

The AM panel of 108 entries was subjected to a post anthesis episode of high temperature stress and/or drought in order to identify MTAs related to wheat's stress response. A high level of heritability was associated with the phenological traits, and with leaf area, peduncle length, plant height, awn length and the number of grains per spike. Grain yield was consistently correlated with flag leaf length, leaf area and peduncle length, under all stress treatment. The outcome of GWAS was the identification of 47 putative stress-specific and 89 involving multiple traits expressed under the various stress treatments, reflecting most probably pleiotropy, but possibly also linkage. Pleiotropic effects are particularly useful in the context of crop improvement, as they allow the breeder to simultaneously select for multiple traits. The pleiotropy/linkage of the loci which contribute both to grain yield and phenology confirms that in a stress-prone environment, early maturity is beneficial for grain filling, as it leads to an increase in grain size and yield. This will add to previously identified genomic regions influencing similar or complementary traits The identification of MTAs which were informative in more than one of the stress treatments suggests a level of shared genetic control of the high temperature and drought stress response. Moreover, the focus of breeding program is to select the genotypes with higher yield at its components under stressed conditions, incorporation of superior alleles through marker assisted selection can be helpful in multi-locus pyramid breeding.

## Supporting information

S1 Table Summary statistics, percent reduction and heritability for agro-physiological traits in CIMMYT wheat lines evaluated in 2 years. (DOCX)

S2 Table Phenotypic correlations based on BLUE values for yield and all other studied traits exposed to [D], [H] stress alone and in combination [HD] for two years. (DOCX)

S3 Table Variance component analysis of traits studied for two seasons (E) under four treatments i.e. non-stressed ([C]), high temperature ([H]), drought ([D]) and combined high temperature and drought ([H+D]) with three independent replications of each. (DOCX)

S4 Table Summary of genome wise and chromosome wise distribution of polymorphic markers. (DOCX)

S5 Table Chromosome wise averaged major allele frequency, polymorphic information content, linkage dis equilibrium and genetic distance. (DOCX)

S6 Table Passport data of wheat lines describing, ID of genotype, origin, sub population and pedigree. (DOCX)

S7 Table Marker trait associations based on BLUE value highlighted MTAs are significant at FDR<0.05 threshold. (XLSX)

S8 Table Comparision of MTAs detected in the present study with previous studies. (XLSX)

S1 Fig Heritability values for all traits under all stress regimes. (TIF)

S2 Fig Neighbour-joining tree based on the Nei’s distance calculated from 9646 polymorphic markers data. (TIF)

S3 Fig Allelic effect size of potent MTAs under four treatments i.e. non-stressed ([C]), high temperature ([H]), drought ([D]) and combined high temperature and drought ([H+D]). (TIF)

S1 File Chromosome-by-chromosome LD plots. (PDF)

S2 File Linkage Mapp of chromosome showing position of significant SNPs and associated traits. (PDF)

S3 File Histograms showing frequency distribution of BLUE values for grain yield across all stress treatments Mannhattan plots and respective quantile qauntile (QQ) plots under control [C]; drought stress [D]; heat stress [H] and combination of heat and drought stress [HD ]. (PPTX)

## Abbrevations

AWL: Awn length
Biomass: Above ground plant dry weight
CIMMYT: International Maize and Wheat Improvement Center
DArT: Density Array Technology
DTA: Days to anthesis
DTH: Days to heading
DTM: Days to maturity
FDR: False discover rate
GPS: Grains per spike
GWAS: Genome wide association studies
GY: Grain yield
HI: Harvest index
LA: Leaf area
LD: linkage disequilibrium
MTAs: Marker Trait Association
PL: Peduncle length
Pext: Peduncle extrusion
PH: Plant height
QTL: Quantitative Trait Loci
RILs: Recombinent inhybrid lines
SLP: Spikelets per spike
SNP: single nucleotide polymorphism
SL: Spike length
Till: Tillers per plant
