# Supplementary material for: Genome-wide association mapping in bread wheat subjected to independent and combined high temperature and drought stress
Source: PLoS One. 2018 Jun 27;13(6):e0199121. doi: 10.1371/journal.pone.0199121 (PMC6021117; doi:10.1371/journal.pone.0199121)
Supplement: S1 Table — (DOCX) [file pone.0199121.s001.docx]

S1_Table: Passport data of wheat lines describing, ID of genotype, origin, sub population and pedigree.

| **Population** | **Genotype ID** | **Origin** | **Cross name** |
| --- | --- | --- | --- |
| pop 1 | 127 | Mexico | FRNCLN*/TECUE #1 |
| pop 1 | 1296 | Mexico | ALD/CEP75630//CEP75234/PT7219/3/BUC/BJY/4/… |
| pop 1 | 6008 | Mexico | ATTILA/3*BCN//BAV92/3/TILHI/5/BAV92/3/PRL/SARA/TSI/VEE#5/4/CROC_1/AE. SQ (224)//2*OPATA |
| pop 1 | 26 | Mexico | CHIR1//CROC/AE. SQ (224) |
| pop 1 | 6070 | Mexico | CNO79//PF70354/MUS/3/PASTOR/4/BAV92*2/5/HAR311 |
| pop 1 | 6071 | Mexico | CROC_1/AE. SQ (224)//OPATA/3/PASTOR |
| pop 1 | 6072 | Mexico | FILIN/3/CROC_1/AE.SQ (205)//KAUZ/4/FILIN |
| pop 1 | 3087 | Mexico | MILAN/KAUZ//PRINIA/3/BAV92/5/TRAP#1/BOW//… |
| pop 1 | 1187 | Mexico | ROLFO7/JUCHI/6/WBLL1/4/HD2281/TRAP#1/3/… |
| pop 1 | 6012 | Mexico | WAXWING*2/SHEILO |
|  |  |  |  |
| pop 2 | 247 | Mexico | ALTAR 84/AE. SQ (219) //2*SERI/3/CHIR 3 |
| pop 2 | Punjab 2011 | Pakistan | AMSEL/ATTILA//INQ-91/PEW'S |
| pop 2 | 107 | Mexico | ATTILA*2/PBW65*2//KACHU |
| pop 2 | Millat 2011 | Pakistan | CHENAB2000/INQ-91 |
| pop 2 | 114 | Mexico | CHIBIA//PRLII/CM65531/3/SKAUZ/BAV92/4/MUNAL #1 |
| pop 2 | Chenab 2008 | Pakistan | CHUM18/BAU |
| pop 2 | 3031 | Mexico | CNO79//PF70354/MUS/3/PASTOR/4/BAV92/5/FRET2/… |
| pop 2 | 517 | Mexico | DANPHE #1*2/CHYAK |
| pop 2 | 522 | Mexico | FRNCLN/3/KIRITATI//HUW234+LR34/PRINIA/4/FRANCOLIN #1 |
| pop 2 | 117 | Mexico | KACHU #1//WBLL1*2/KUKUNA |
| pop 2 | 115 | Mexico | KACHU//WBLL1*2/BRAMBLING |
| pop 2 | 4422 | Mexico | KAUZ/PASTOR//PBW343/3/KIRITATI/4/FRNCLN |
| pop 2 | 125 | Mexico | KIRITATI/WBLL1//MESIA/3/KIRITATI/WBLL1 |
| pop 2 | 131 | Mexico | MILAN/S87230/BAV92*2/3/AKURI |
| pop 2 | 528 | Mexico | MUNAL*2/CHONTE |
| pop 2 | 4424 | Mexico | ND643/2*TRCH//MUTUS/4/PFAU/SERI.1B//AMAD/3/….. |
| pop 2 | 509 | Mexico | ND643/2*WBLL1/4/WHEAR/KAKUNA/3/C80.1/3*BATAVIA//2*WBLL1 |
| pop 2 | 1290 | Mexico | PBW343*2/KUKUNA*2//WHEAR |
| pop 2 | 6075 | Mexico | PFAU/SERI.1B//AMAD/3/WAXING/4/BAJ #1 |
| pop 2 | 4423 | Mexico | PFAU/SERI.1B//AMAD/3/WAXWING*2/4/TECUE #1 |
| pop 2 | 504 | Mexico | PRL/2*PASTOR/3PFAU/WEAVER*2//CHAPIO |
| pop 2 | 527 | Mexico | PRL/2*PASTOR/4/CHOIX/STAR/3/HE1/3*CNO79//2*SERI/5/… |
| pop 2 | 108 | Mexico | ROLF07/4/BOW/NKT//CBRD/3/CBRD/5/FRET2/TUKURU//FRET2 |
| pop 2 | 1112 | Mexico | STLN/MUNAL #1 |
| pop 2 | 505 | Mexico | TACUPETO F2001*2/BRAMBLING//KIRITATI/2*TRCH |
| pop 2 | 130 | Mexico | WBLL1*2/VIVITSI//AKURI/3/WBLL1*2/BRAMBLING |
| pop 2 | 512 | Mexico | WHEAR/KUKUNA/3/C80.1/3*BATAVIA//2*WBLL1/5/PRL/2*PASTOR/.. |
| pop 2 | 513 | Mexico | WHEAR/VIVITSI//WHEAR/3/FRNCLN |
| pop 2 | Galaxy 2013 | Pakistan |  |
|  |  |  |  |
| pop3 | Chakwal 50 | Pakistan | ATTILA/3/HUI/CARC//CHEN/CHTO/4/ATTILA |
| pop3 | 27 | Mexico | C80.1/3*QT4118//KAUZ/RAYON/3/2*TARCH/4/BERKUT/KRICHAUFF |
| pop3 | 268 | Mexico | DUCULA//HUI/TUB/3/CAZO/4/CROC-1/AE. SQ (224)//OPATA |
| pop3 | 186 | Mexico | DVERD-2/AE. SQ (214)//2*ESDA/3/NS732/HER |
| pop3 | 6025 | Mexico | FRET2*2/KUKUNA*2/4/BOW/URES//2*WEAVER/3/CROC_1/AE. SQ (213)//PGO |
| pop3 | 3038 | Mexico | GK ARON/AG SECO7846//2180/4/2*MILAN/KAUZ//… |
| pop3 | 3059 | Mexico | GONDO//WBLL1*2/TUKURU/4/GONDO//SHA5/WEAVER/… |
| pop3 | 3051 | Mexico | GOUBARA-1/2*SOKOLL |
| pop3 | Durabi 2011 | Pakistan | HXL7573/2*BAU//PASTOR |
| pop3 | 6049 | Mexico | INQLAB 91*2/TUKURU//WHEAR |
| pop3 | 126 | Mexico | KIRITATI/WBLL1/4/2*BABAX/LR42//BABAX*2/3/KURUKU |
| pop3 | 6017 | Mexico | KSW/SAUAL//SAUAL |
| pop3 | 519 | Mexico | MUTUS*2//ND643/2*WBLL1 |
| pop3 | 113 | Mexico | NAC/TH.AC//3*PVN/3/MIRLO/BUC/4/2*PASTOR/5/KACHU/6/KACHU |
| pop3 | Narc 2011 | Pakistan | OASIS/SKAUZ//4*BCN/3/2*PASTOR |
| pop3 | 3134 | Mexico | PASTOR*2/BAV92/3/FRET2/KUKUNA//FRET2 |
| pop3 | 34 | Mexico | PASTOR//HXL7573/2*BAU/3/WBLL1 |
| pop3 | Faislabad 2008 | Pakistan | PBW65/2*Pastor |
| pop3 | 4402 | Mexico | PRL/2*PASTOR |
| pop3 | 4427 | Mexico | PWB65/2*PATOR/3/KIRTATI//ATTILA*2/PASTOR |
| pop3 | 78 | Mexico | SABUF//ALTAR84/AE. SQ (205) |
| pop3 | 110 | Mexico | SAUAL/3/ACHTAR*3//KANZ/KS85-8-4/4/SAUAL |
| pop3 | 3052 | Mexico | SOKOL*2/3/PASTOR//MUNIA/ALTAR84 |
| pop3 | 25 | Mexico | SOKOLL//PUB94.15.1.12/WBLL1 |
| pop3 | 37 | Mexico | SOKOLL/WBLL1 |
| pop3 | Miraj 2008 | Pakistan | SPARROW/INIA//V.7394/WL711/13/BAUS |
| pop3 | 3017 | Mexico | VORB/3/T.DICOCCON PI94625/AR.SAQUARROSA (372)…… |
| pop3 | 3014 | Mexico | VORB/SOKOLL |
| pop3 | Pak 2013 | Pakistan |  |
|  |  |  |  |
| pop4 | 511 | Mexico | BAJ #1/KISKADEE #1 |
| pop4 | 525 | Mexico | CROC_1/AE.SAQUARROSA (205)//BORL95/3/PRL/SARA//TSI/….. |
| pop4 | 526 | Mexico | FRET2*2/4/SNI/TRAP#1/3/KAZ*2/TRAP//KAUZ/5/KIRITATI/….. |
| pop4 | 1211 | Mexico | FRET2/KUKUNA//FRET2/3/PASTOR//HXL7573/2*BAU/… |
| pop4 | 506 | Mexico | KACHU//KIRITATI/2*TRCH |
| pop4 | 507 | Mexico | KIRITATI//HAW234+LR34/PRINIA/3/BAJ #1 |
| pop4 | 518 | Mexico | MUTUS*2/HARIL #1 |
| pop4 | 515 | Mexico | MUU/FRNCLN//FRANCOLIN #1 |
| pop4 | 4503 | Mexico | PANDORA//WEBLL1*2/BRAMBLING/3/WBLL1*2/….. |
| pop4 | 6113 | Mexico | PASTOR/3/ALTAR84/AE.SQ // OPATA |
| pop4 | 128 | Mexico | PFAU/SERI.1B//AMAD/3/WAXWING/4/AKURI/5/PFAU/SERI.1B//….. |
| pop4 | 119 | Mexico | PFAU/SERI.1B//AMAD/3/WAXWING/4/WBLL1*2/BRAMBLING |
| pop4 | 1295 | Mexico | PSN/BOW//SERI/3/MILAN/4/ATTILA/5/KAUZ*2/… |
| pop4 | 524 | Mexico | SWSR22T.B./2*BLOUK #1//WBLL1*2/KURUKU |
| pop4 | 6024 | Mexico | TRCH/5/REH/HARE//2*BCN/3/CROC_1/AE. SQ (213)//PGO_4/HUITES |
| pop4 | 523 | Mexico | WBLL1*2BRAMBLING*2//BAVIS |
| pop4 | 123 | Mexico | WBLL4/KUKUNA//WBLL1*2/BRAMBLING |
| pop4 | 503 | Mexico |  |
|  |  |  |  |
| pop 5 | 112 | Mexico | ALTAR 84/AE.SQUARROSA (221)//3*BORL95/3/URES/JUN/KAZU/… |
| pop 5 | 13 | Mexico | ATTILA/5/CHIR3/4/SIREN//ALTAR 84/AE. SQ (205)/3/3*BUC/6/FCT |
| pop 5 | 3056 | Mexico | BOW/VEE/5/ND/VG9144//KAL/BB/3/YACO/4/CHIL/6/… |
| pop 5 | 4419 | Mexico | FRET*2/BRAMBLING//BECARD/3/WBLL1*2/…. |
| pop 5 | 520 | Mexico | FRNCLN/NIINI #1//FRANCOLIN #1 |
| pop 5 | 6006 | Mexico | GAN/AE.SQUARROSA (408)//2*OASIS/5*BORL95/3/……. |
| pop 5 | 124 | Mexico | ITP40/AKURI |
| pop 5 | 4421 | Mexico | KACHU/BECARD//WBLL1*2/BRAMBLING |
| pop 5 | 134 | Mexico | KACHU/KINDE |
| pop 5 | 116 | Mexico | KACHU/KIRITATI |
| pop 5 | 118 | Mexico | KIRITATI/WBLL1//FARNCOLIN #1 |
| pop 5 | 150 | Mexico | ND643/2*TRCH/3/MILAN/S87230//BAV92/4/PFAU/SERI.1B//… |
| pop 5 | 6039 | Mexico | ONIX/KBIRD |
| pop 5 | 135 | Mexico | PBW343*2/KUKUNA/3/PASTOR//CHIL/PRL/4/GRACK |
| pop 5 | 514 | Mexico | QUAIU*2/KINDE |
| pop 5 | 6011 | Mexico | ROLFO7*2/5/FCT/3/GOV/AZ//MUS/4/DOVE/BUC |
| pop 5 | 19 | Mexico | SERI/BAV92//PUB94.15.1.12/WBLL1 |
| pop 5 | 3013 | Mexico | W15.92/4/PASTOR//HXL7573/2*BAU/3/WBLL1 |
| pop 5 | 529 | Mexico | WAXWING*2/TUKURU/2*FRNCLN |
| pop 5 | 516 | Mexico | WAXWING*2/TUKURU/3/2*WHEAR/VIVITSI//WHEAR |
| pop 5 | 4417 | Mexico | WBLL1*2/BRAMBLING//CHYAK |
| pop 5 | 16 | Mexico | WBLL1//PUB94.15.1.12/WBLL1 |
