# Supplementary material for: Genome-wide association mapping in bread wheat subjected to independent and combined high temperature and drought stress
Source: PLoS One. 2018 Jun 27;13(6):e0199121. doi: 10.1371/journal.pone.0199121 (PMC6021117; doi:10.1371/journal.pone.0199121)
Supplement: S2 Table — (DOCX) [file pone.0199121.s002.docx]

S2_Table. Summary statistics, percent reduction and heritability for yield and related traits in CIMMYT wheat lines evaluated in 2 years

| **Trait** | **Treatment** | **Min** | **Max** | **Mean** | **S.E** | **LSD** | **%CV** | **% reduction** | **H^2^** |
| --- | --- | --- | --- | --- | --- | --- | --- | --- | --- |
| **Awn Length** | **Control** | 3.08 | 10.98 | 6.86 | 0.20 | 0.39 | 29.72 |  | 0.89 |
|  | **Drought** | 2.12 | 8.80 | 4.43 | 0.17 | 0.34 | 39.81 | -35.41 | 0.84 |
|  | **Heat** | 1.33 | 6.92 | 4.72 | 0.15 | 0.31 | 34.07 | -31.25 | 0.92 |
|  | **Combination** | 1.17 | 7.00 | 4.25 | 0.13 | 0.25 | 30.97 | -37.99 | 0.90 |
| **Biomass** | **Control** | 37.00 | 79.67 | 47.75 | 0.97 | 1.92 | 21.04 |  | 0.69 |
|  | **Drought** | 23.00 | 49.33 | 37.97 | 0.68 | 1.34 | 18.54 | -20.47 | 0.78 |
|  | **Heat** | 24.67 | 54.51 | 40.62 | 0.63 | 1.26 | 16.23 | -14.92 | 0.91 |
|  | **Combination** | 15.67 | 39.71 | 28.63 | 0.51 | 1.01 | 18.45 | -40.05 | 0.70 |
| **Days to anthesis** | **Control** | 118.34 | 129.00 | 125.11 | 0.20 | 0.39 | 1.65 |  | 0.35 |
|  | **Drought** | 109.50 | 126.50 | 116.18 | 0.43 | 0.86 | 3.88 | -7.14 | 0.91 |
|  | **Heat** | 101.00 | 120.00 | 110.95 | 0.36 | 0.72 | 3.41 | -11.32 | 0.73 |
|  | **Combination** | 98.00 | 110.50 | 105.00 | 0.26 | 0.52 | 2.57 | -16.07 | 0.89 |
| **Days to heading** | **Control** | 102.00 | 113.50 | 108.34 | 0.25 | 0.49 | 2.38 |  | 0.41 |
|  | **Drought** | 87.00 | 115.00 | 106.44 | 0.48 | 0.95 | 4.69 | -1.76 | 0.93 |
|  | **Heat** | 93.00 | 108.50 | 102.40 | 0.29 | 0.58 | 2.95 | -5.49 | 0.73 |
|  | **Combination** | 83.00 | 97.50 | 92.56 | 0.27 | 0.54 | 3.03 | -14.57 | 0.92 |
| **Days to maturity** | **Control** | 147.00 | 168.00 | 158.55 | 0.57 | 1.12 | 3.70 |  | 0.91 |
|  | **Drought** | 128.00 | 155.00 | 145.33 | 0.62 | 1.23 | 4.43 | -8.34 | 0.95 |
|  | **Heat** | 129.00 | 153.50 | 140.02 | 0.48 | 0.95 | 3.57 | -11.69 | 0.84 |
|  | **Combination** | 118.00 | 139.00 | 128.21 | 0.43 | 0.84 | 3.45 | -19.14 | 0.89 |
| **Grains per spike** | **Control** | 59.16 | 106.33 | 75.44 | 0.94 | 1.87 | 12.98 |  | 0.86 |
|  | **Drought** | 41.00 | 87.17 | 63.60 | 1.09 | 2.17 | 17.88 | -15.68 | 0.70 |
|  | **Heat** | 34.17 | 90.67 | 67.87 | 1.16 | 2.29 | 17.70 | -10.03 | 0.81 |
|  | **Combination** | 22.00 | 79.67 | 55.44 | 1.34 | 2.65 | 25.02 | -26.50 | 0.79 |
| **Grain yield** | **Control** | 17.40 | 43.95 | 26.02 | 0.59 | 1.17 | 23.57 |  | 0.83 |
|  | **Drought** | 5.44 | 29.99 | 14.40 | 0.51 | 1.01 | 36.74 | -44.66 | 0.68 |
|  | **Heat** | 7.73 | 29.58 | 12.21 | 0.36 | 0.72 | 30.70 | -53.05 | 0.67 |
|  | **Combination** | 5.08 | 13.97 | 11.33 | 0.17 | 0.34 | 15.52 | -56.47 | 0.88 |
| **Harvest index** | **Control** | 37.33 | 93.40 | 57.82 | 1.30 | 2.58 | 23.43 |  | 0.69 |
|  | **Drought** | 20.71 | 54.65 | 36.23 | 0.77 | 1.53 | 22.11 | -37.35 | 0.65 |
|  | **Heat** | 23.18 | 50.50 | 35.02 | 0.63 | 1.24 | 18.57 | -39.44 | 0.64 |
|  | **Combination** | 14.21 | 49.16 | 34.05 | 0.69 | 1.37 | 21.17 | -41.12 | 0.80 |
| **Leaf area** | **Control** | 38.39 | 72.86 | 47.68 | 0.69 | 1.38 | 15.14 |  | 0.76 |
|  | **Drought** | 22.97 | 60.35 | 35.44 | 0.62 | 1.24 | 18.28 | -25.68 | 0.92 |
|  | **Heat** | 19.38 | 47.20 | 34.73 | 0.70 | 1.40 | 21.08 | -27.15 | 0.93 |
|  | **Combination** | 16.18 | 37.98 | 29.58 | 0.48 | 0.96 | 17.04 | -37.97 | 0.95 |
| **Peduncle length** | **Control** | 35.33 | 47.90 | 40.85 | 0.30 | 0.60 | 7.64 |  | 0.85 |
|  | **Drought** | 22.52 | 40.32 | 32.12 | 0.42 | 0.83 | 13.47 | -21.37 | 0.80 |
|  | **Heat** | 25.38 | 41.77 | 33.96 | 0.40 | 0.79 | 12.27 | -16.88 | 0.85 |
|  | **Combination** | 18.05 | 31.78 | 25.18 | 0.33 | 0.66 | 13.77 | -38.38 | 0.68 |
| **Peduncle extrusion** | **Control** | 5.09 | 26.97 | 14.03 | 0.47 | 0.92 | 34.45 |  | 0.93 |
|  | **Drought** | 3.17 | 16.22 | 10.80 | 0.25 | 0.50 | 24.16 | -23.05 | 0.90 |
|  | **Heat** | 6.48 | 19.48 | 10.67 | 0.31 | 0.61 | 30.13 | -24.00 | 0.88 |
|  | **Combination** | 5.17 | 13.90 | 9.67 | 0.17 | 0.33 | 17.73 | -31.08 | 0.93 |
| **Plant height** | **Control** | 72.50 | 104.83 | 88.23 | 0.62 | 1.22 | 7.28 |  | 0.94 |
|  | **Drought** | 59.17 | 110.50 | 81.25 | 0.91 | 1.80 | 11.63 | -7.91 | 0.90 |
|  | **Heat** | 65.55 | 92.50 | 77.59 | 0.59 | 1.17 | 7.92 | -12.06 | 0.88 |
|  | **Combination** | 51.83 | 77.67 | 63.65 | 0.58 | 1.14 | 9.41 | -27.87 | 0.89 |
| **Spikelets per spike** | **Control** | 17.00 | 24.00 | 21.09 | 0.14 | 0.28 | 6.87 |  | 0.38 |
|  | **Drought** | 16.67 | 23.30 | 19.38 | 0.14 | 0.27 | 7.30 | -8.10 | 0.58 |
|  | **Heat** | 15.33 | 24.00 | 19.14 | 0.16 | 0.31 | 8.54 | -9.28 | 0.62 |
|  | **Combination** | 10.33 | 22.67 | 18.09 | 0.24 | 0.47 | 13.53 | -14.22 | 0.64 |
| **Spike length** | **Control** | 9.28 | 20.00 | 13.61 | 0.25 | 0.50 | 19.11 |  | 0.68 |
|  | **Drought** | 6.33 | 16.92 | 11.81 | 0.22 | 0.44 | 19.55 | -13.23 | 0.91 |
|  | **Heat** | 6.05 | 15.72 | 10.33 | 0.20 | 0.40 | 20.50 | -24.10 | 0.87 |
|  | **Combination** | 7.53 | 12.15 | 9.51 | 0.10 | 0.20 | 10.92 | -30.09 | 0.82 |
| **Tillers per plant** | **Control** | 6.00 | 10.83 | 9.40 | 0.11 | 0.21 | 11.97 |  | 0.37 |
|  | **Drought** | 3.17 | 10.17 | 7.69 | 0.15 | 0.30 | 20.26 | -18.19 | 0.59 |
|  | **Heat** | 3.00 | 9.50 | 6.59 | 0.15 | 0.29 | 23.00 | -29.94 | 0.82 |
|  | **Combination** | 1.33 | 4.00 | 3.95 | 0.17 | 0.23 | 27.46 | -37.98 | 0.60 |
