# Supplementary material for: Genome-wide association mapping in bread wheat subjected to independent and combined high temperature and drought stress
Source: PLoS One. 2018 Jun 27;13(6):e0199121. doi: 10.1371/journal.pone.0199121 (PMC6021117; doi:10.1371/journal.pone.0199121)
Supplement: S3 Table — (DOCX) [file pone.0199121.s003.docx]

S3_Table: Phenotypic correlations of yield with all other studied traits based on BLUE values exposed to [C], [D], [H] stress alone and in combination [HD] for two years.

| **Trait** | **Control** | **Drought** | **Heat** | **Combination** |
| --- | --- | --- | --- | --- |
| AWL | -0.14 | 0.53** | 0.08 | 0.22* |
| Biomass | 0.36** | 0.51** | 0.16 | -0.24* |
| DTA | -0.61** | 0.46** | -0.07 | 0.11 |
| DTH | 0.55** | 0.49** | 0.14** | 0.61** |
| DTM | -0.54** | 0.56** | 0.14 | -0.58** |
| GPS | -0.13 | 0.38** | -0.06 | 0.22* |
| HI | 0.58** | 0.68** | 0.07 | 0.52** |
| LA | 0.24* | 0.24* | -0.09 | 0.55** |
| PL | 0.40** | 0.36** | 0 | 0.43** |
| Pext | -0.06 | 0.40** | -0.28** | 0.70** |
| PH | 0.64** | 0.63** | 0.17 | 0.40** |
| SLPs | -0.11 | 0.66** | 0.32** | 0.09 |
| SPL | 0.37** | 0.65** | -0.34** | -0.27** |
| Till | 0.58** | 0.61** | -0.01 | 0.53** |

AWL: Awn length, Biomass: Above ground plant dry weight, DTA: Days to anthesis, DTH: Days to heading, DTM: Days to maturity, GPS: Grains per spike, GY: Grain yield, HI: Harvest index, LA: Leaf area, PL: Peduncle length, Pext: Peduncle extrusion, PH: Plant height, SPLs: Spikelets per spike, SPL: Spike length, Till: Tillers per plant,
