# Supplementary material for: Genome-wide association mapping in bread wheat subjected to independent and combined high temperature and drought stress
Source: PLoS One. 2018 Jun 27;13(6):e0199121. doi: 10.1371/journal.pone.0199121 (PMC6021117; doi:10.1371/journal.pone.0199121)
Supplement: S4 Table — (DOCX) [file pone.0199121.s004.docx]

S4_Table: Variance component analysis of traits studied for two seasons (E) under four treatments i.e. non-stressed ([C]), high temperature ([H]), drought ([D]) and combined high temperature and drought ([H+D]) with three independent replications of each.

| Traits | **Treatment** | **Genotype** | **Environment** | **Treatment**$\boldsymbol{\times}$  **Genotype** | **Genotype**$\boldsymbol{\times}$  **Environment** | **Treatment**$\boldsymbol{\times}$  **Environment** | **Treatment**$\boldsymbol{\times}$  **Genotype**$\boldsymbol{\times}$  **Environment** | **Residuals** |
| --- | --- | --- | --- | --- | --- | --- | --- | --- |
| AWL | 16.9*** | 9.84*** | 54.25*** | 2.79*** | 0.37 | 6.39*** | 0.42 | 0.76 |
| BIOMASS | 23282*** | 207*** | 350*** | 48*** | 17 | 53 | 15 | 31 |
| DTH | 1915.6*** | 34*** | NA | 6.7*** | NA | NA | NA | 0.3 |
| DTA | 2310.7*** | 31*** | NA | 6.6*** | NA | NA | NA | 0.5 |
| DTM | 2112.7*** | 31*** | NA | 6.6ns | NA | NA | NA | 11.2 |
| GPS | 87569*** | 785*** | 11965*** | 136*** | 104* | 1151*** | 53 | 82 |
| GY | 6259*** | 75*** | 1653*** | 22*** | 13*** | 586*** | 12*** | 6 |
| HI | 9853*** | 777*** | 10907*** | 208*** | 119 | 5558*** | 129** | 101 |
| LA | 17282*** | 743*** | 480*** | 50*** | 55*** | 13 | 7 | 34 |
| PEXT | 40.76*** | 89.45*** | 205.59*** | 26.82*** | 2.39ns | 30.44*** | 1.77 | 4.79 |
| PDL | 665.5*** | 155.7*** | 1000*** | 74.5*** | 5.3 | 112.9*** | 4.8 | 10.4 |
| PH | 4247*** | 370*** | 1380*** | 100*** | 15ns | 116*** | 8 | 17 |
| SPL | 2.25ns | 17.91*** | 165.01*** | 3.89*** | 0.65ns | 15.74*** | 0.64 | 1.5 |
| SPLS | 70.36*** | 29.28*** | 0.1ns | 8.96*** | 0.06ns | 0.09 | 0.24 | 1.06 |
| TILL | 888.9*** | 30.8*** | 1ns | 8.4*** | 1.4ns | 269.5*** | 7.2*** | 2.8 |
