# Supplementary material for: Genome-wide association mapping in bread wheat subjected to independent and combined high temperature and drought stress
Source: PLoS One. 2018 Jun 27;13(6):e0199121. doi: 10.1371/journal.pone.0199121 (PMC6021117; doi:10.1371/journal.pone.0199121)
Supplement: S5 Table — (DOCX) [file pone.0199121.s005.docx]

S5_Table: Summary of genome wise and chromosome wise distribution of polymorphic markers

| **Chromosome** | **A** | **B** | **D** | **Total by Group** |
| --- | --- | --- | --- | --- |
| 1 | 362 | 703 | 251 | **1316** |
| 2 | 469 | 907 | 337 | **1713** |
| 3 | 427 | 614 | 107 | **1148** |
| 4 | 342 | 263 | 42 | **647** |
| 5 | 522 | 766 | 86 | **1374** |
| 6 | 566 | 685 | 124 | **1375** |
| 7 | 590 | 591 | 103 | **1284** |
| **Total by Genome** | **3278** | **4529** | **1050** | **8857** |
| **unmapped** |  |  |  | **789** |
| **Total** |  |  |  | **9646** |
