# Supplementary material for: Genome-wide association mapping in bread wheat subjected to independent and combined high temperature and drought stress
Source: PLoS One. 2018 Jun 27;13(6):e0199121. doi: 10.1371/journal.pone.0199121 (PMC6021117; doi:10.1371/journal.pone.0199121)
Supplement: S6 Table — (DOCX) [file pone.0199121.s006.docx]

S6_Table: Chromosome wise averaged major allele frequency, polymorphic information content, linkage dis equilibrium and genetic distance.

| **Chromosome** | **Mean PIC** | **Major Allele Frequency** | **Mean Distance** | **Mean LD** |
| --- | --- | --- | --- | --- |
| 1A | 0.31 | 0.7 | 11.81 | 0.45 |
| 1B | 0.26 | 0.75 | 7.97 | 0.39 |
| 1D | 0.25 | 0.76 | 51.95 | 0.49 |
| 2A | 0.3 | 0.72 | 10.19 | 0.41 |
| 2B | 0.29 | 0.72 | 3.28 | 0.38 |
| 2D | 0.24 | 0.81 | 5.55 | 0.38 |
| 3A | 0.25 | 0.78 | 4.96 | 0.48 |
| 3B | 0.25 | 0.78 | 7.19 | 0.41 |
| 3D | 0.21 | 0.82 | 6.53 | 0.42 |
| 4A | 0.27 | 0.74 | 4.71 | 0.47 |
| 4B | 0.27 | 0.76 | 3.19 | 0.44 |
| 4D | 0.3 | 0.68 | 2.57 | 0.64 |
| 5A | 0.28 | 0.73 | 16.50 | 0.38 |
| 5B | 0.28 | 0.72 | 7.44 | 0.42 |
| 5D | 0.22 | 0.81 | 3.97 | 0.50 |
| 6A | 0.26 | 0.76 | 6.23 | 0.40 |
| 6B | 0.31 | 0.71 | 3.44 | 0.42 |
| 6D | 0.3 | 0.7 | 3.03 | 0.53 |
| 7A | 0.29 | 0.71 | 4.65 | 0.46 |
| 7B | 0.29 | 0.72 | 5.25 | 0.39 |
| 7D | 0.29 | 0.71 | 1.52 | 0.60 |
| ump | 0.24 | 0.79 |  |  |
