# Supplementary figures and images for: Genome-wide association mapping in bread wheat subjected to independent and combined high temperature and drought stress
Source: PLoS One. 2018 Jun 27;13(6):e0199121. doi: 10.1371/journal.pone.0199121 (PMC6021117; doi:10.1371/journal.pone.0199121)

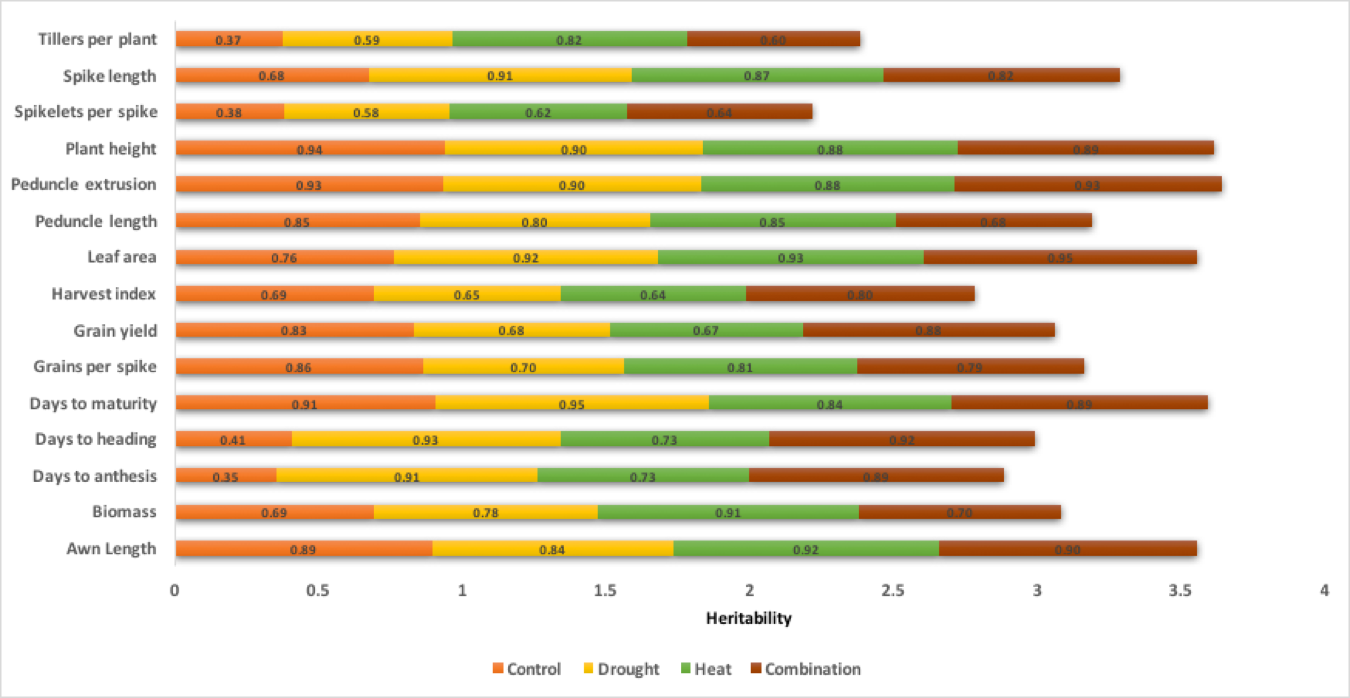

Supplement: S1 Fig — (TIF) [file pone.0199121.s009.tif]

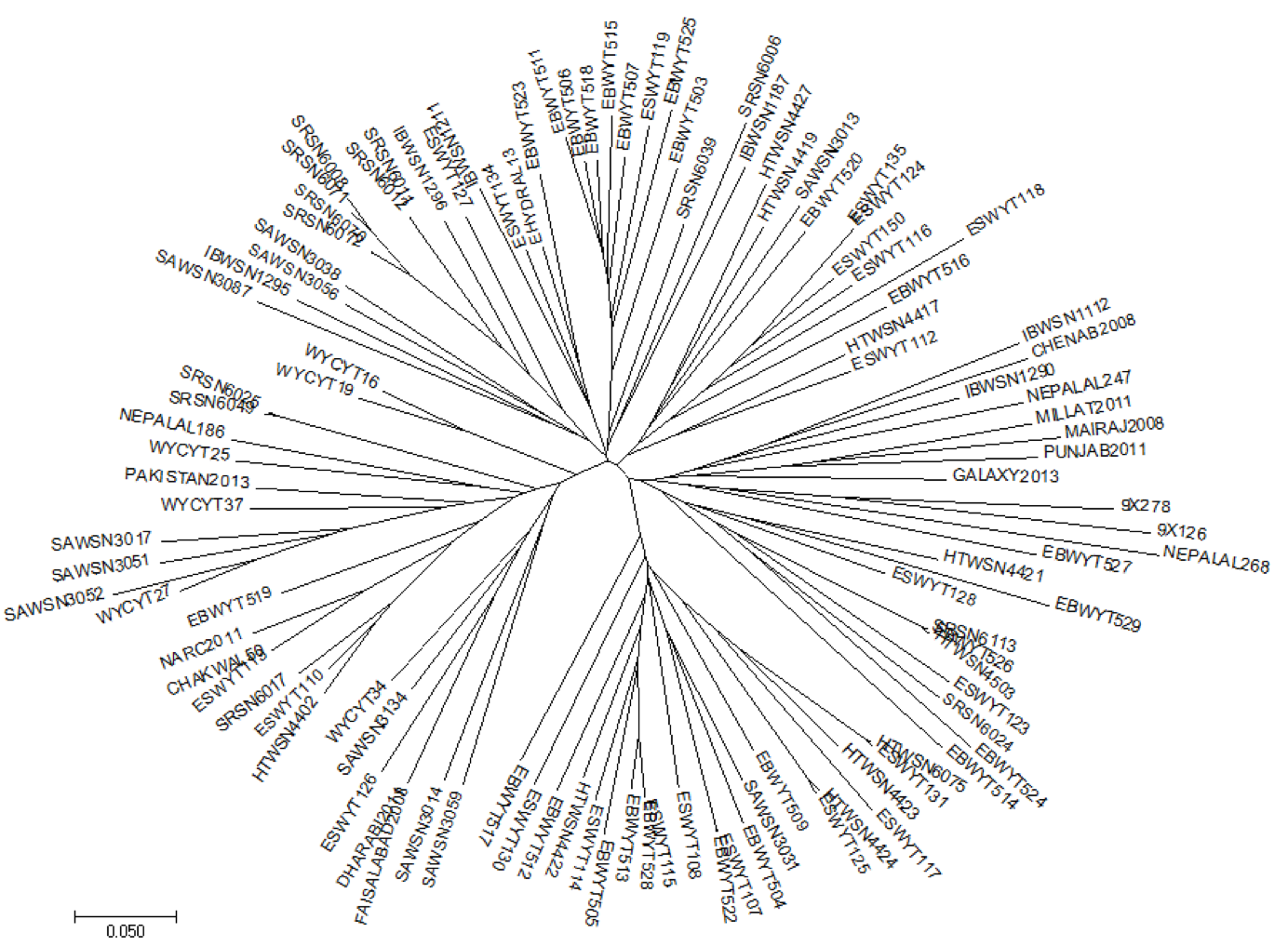

Supplement: S2 Fig — (TIF) [file pone.0199121.s010.tif]

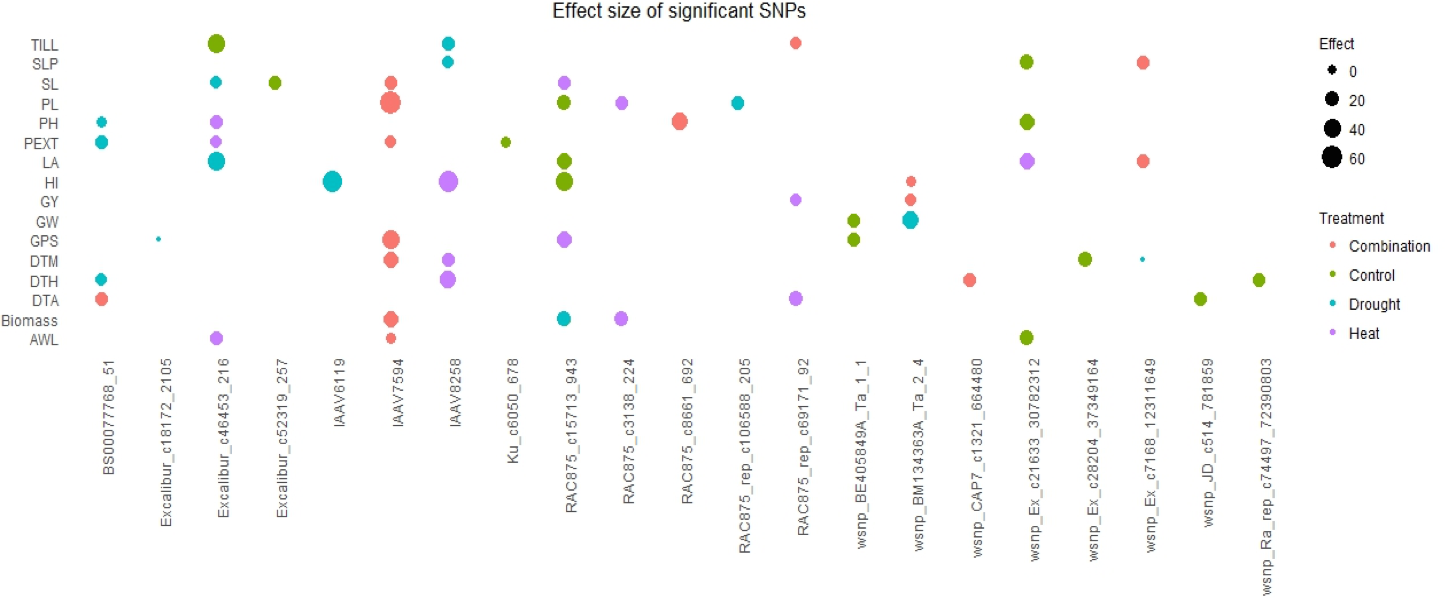

Supplement: S3 Fig — (TIF) [file pone.0199121.s011.tif]

## Slide 1
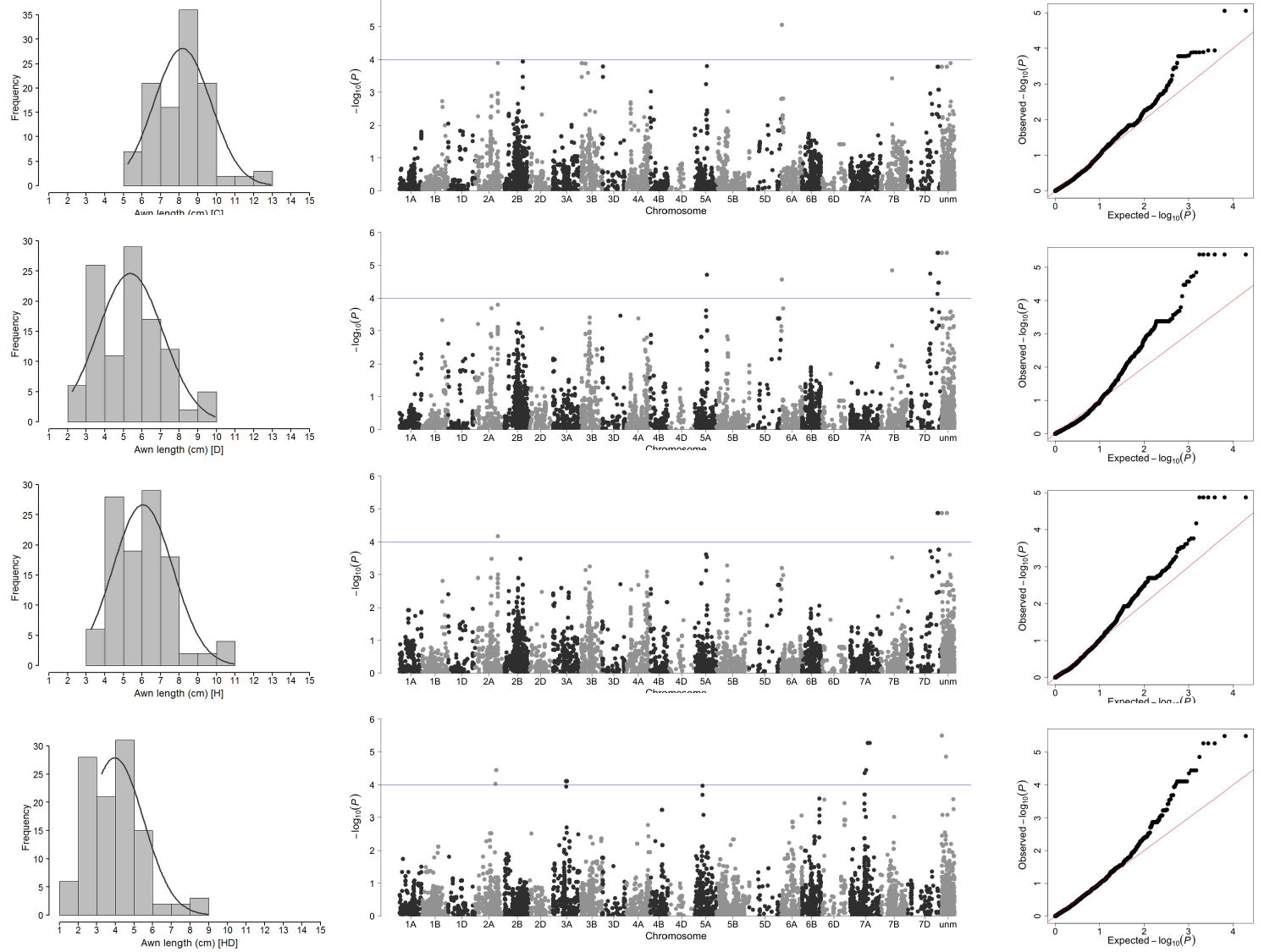

## Slide 2
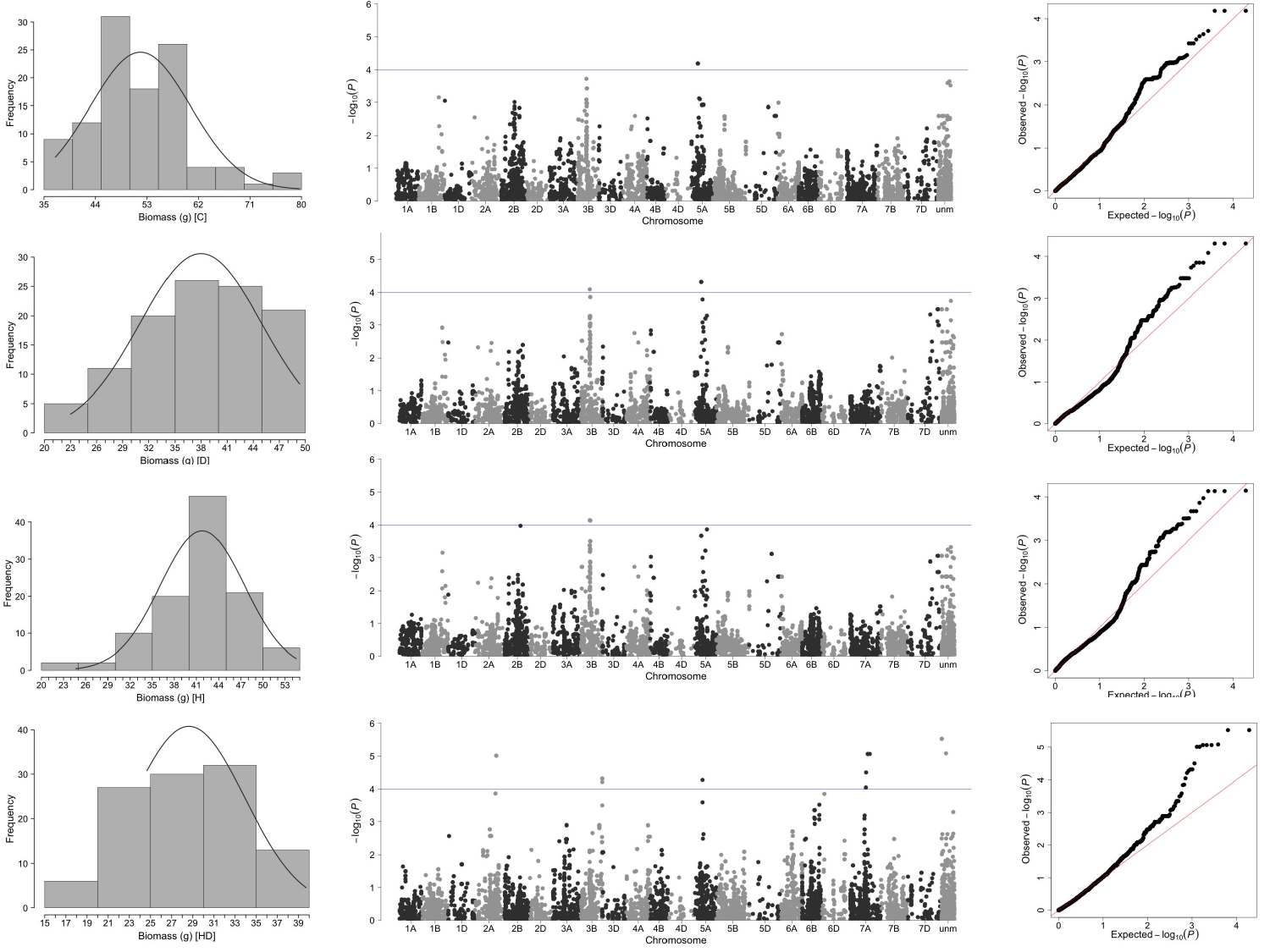

## Slide 3
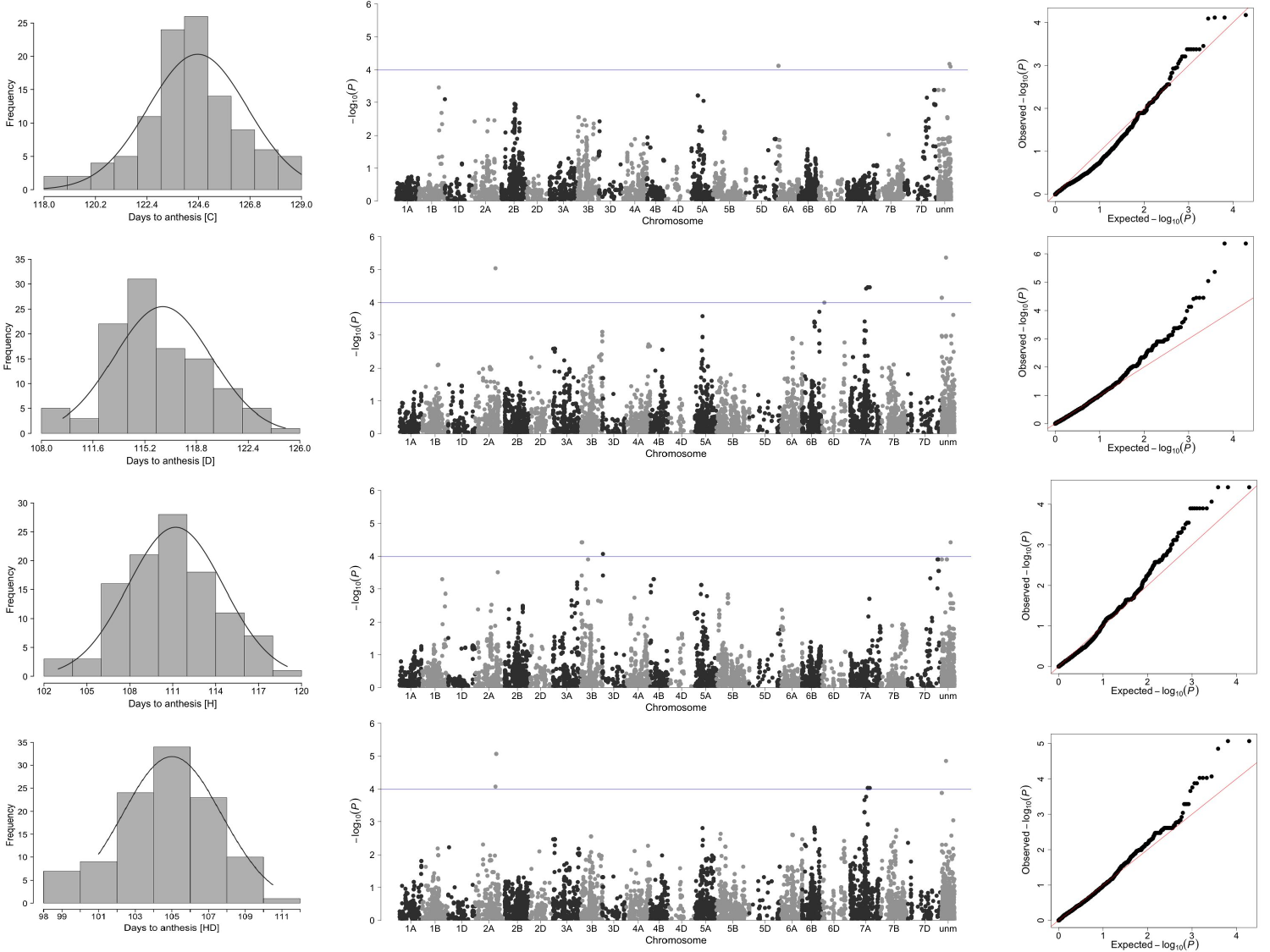

## Slide 4
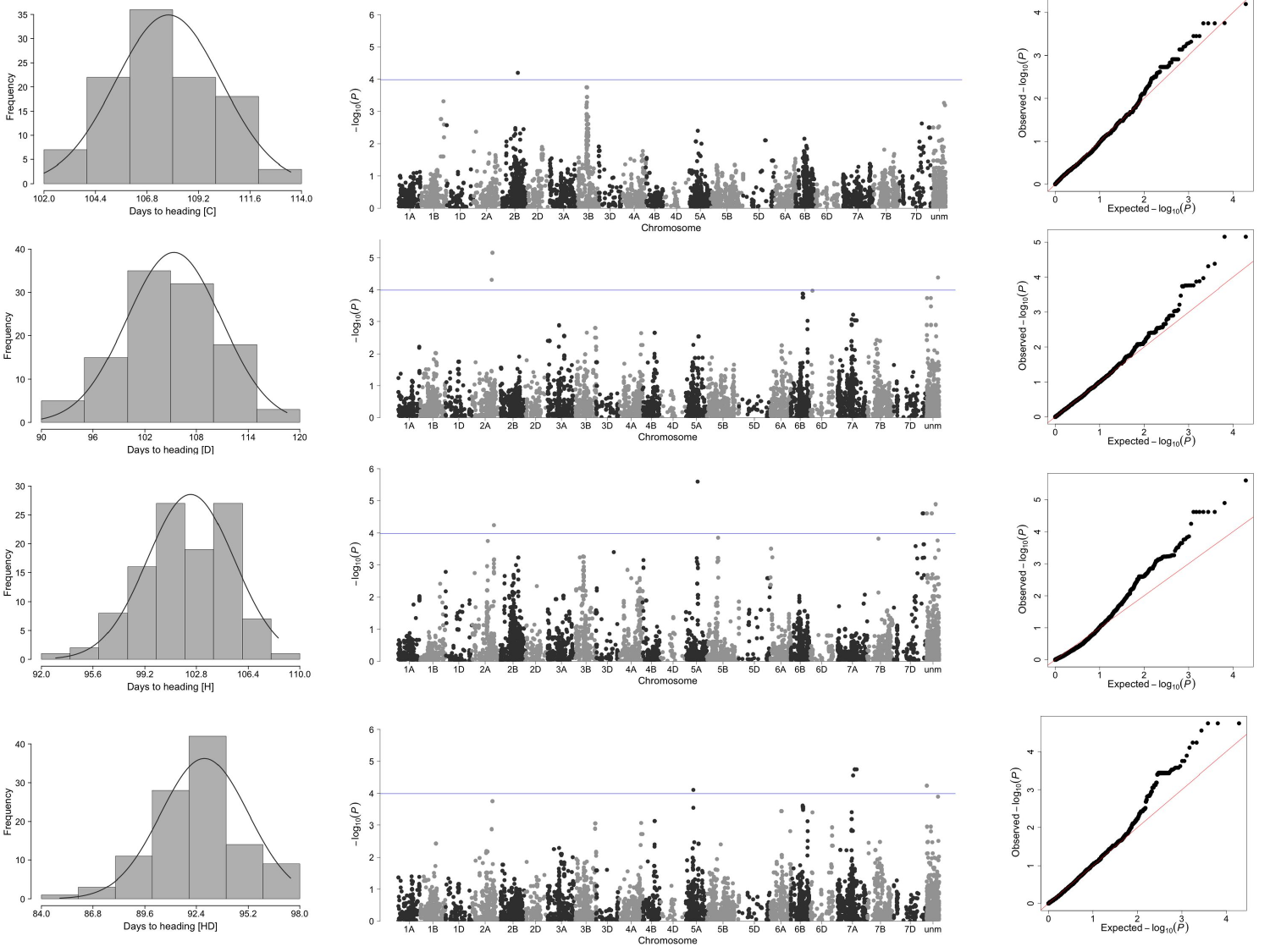

## Slide 5
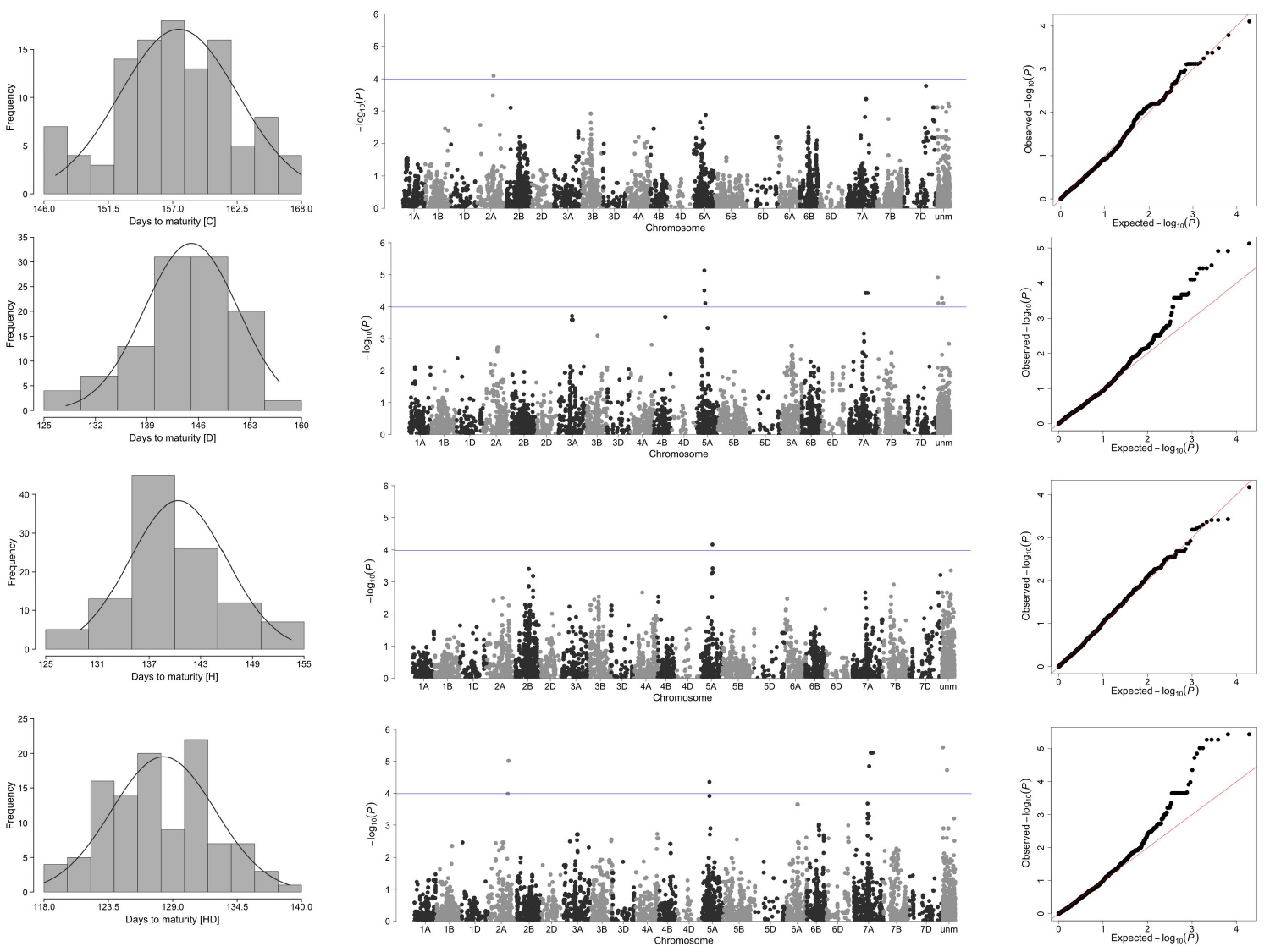

## Slide 6
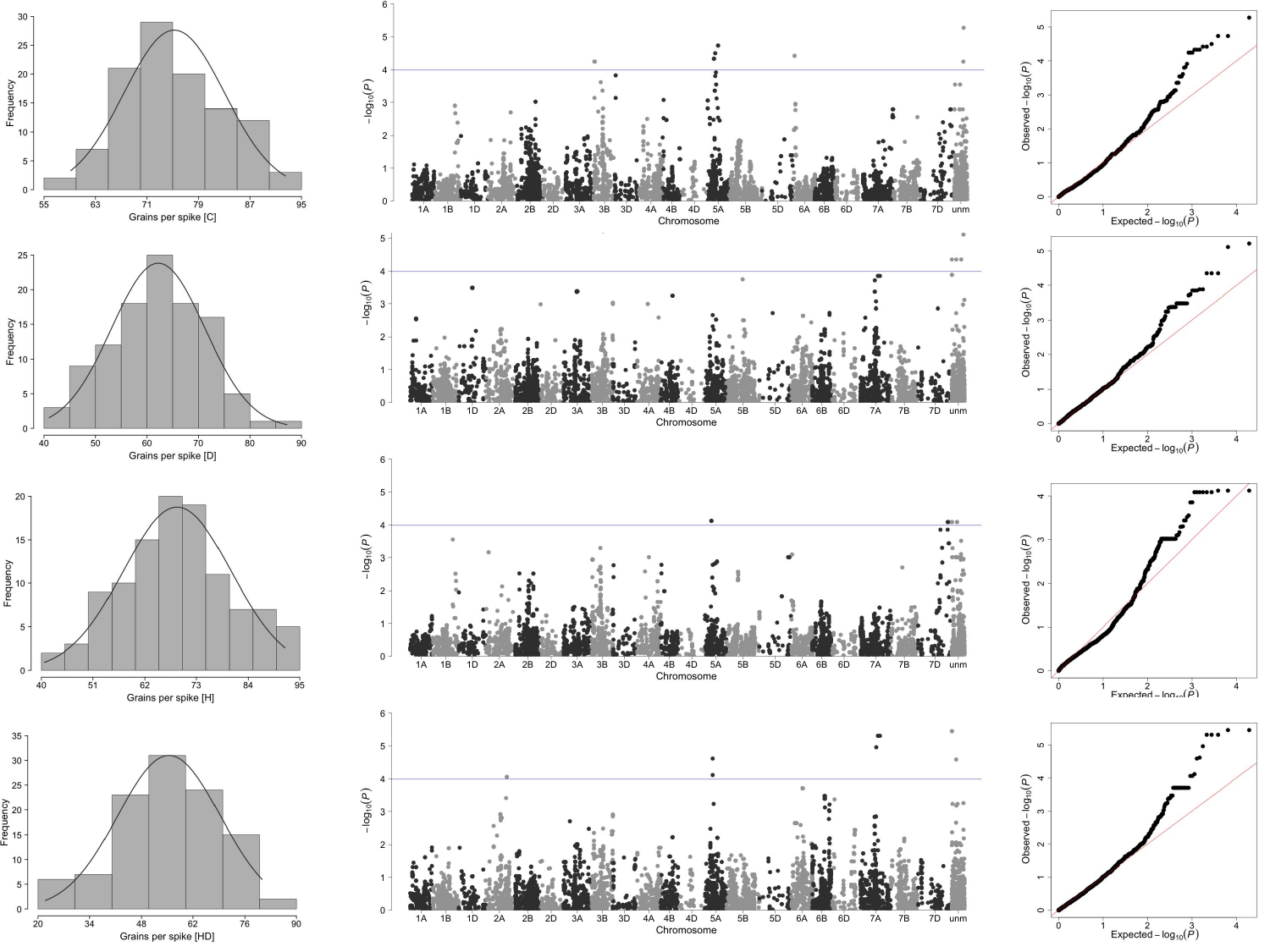

## Slide 7
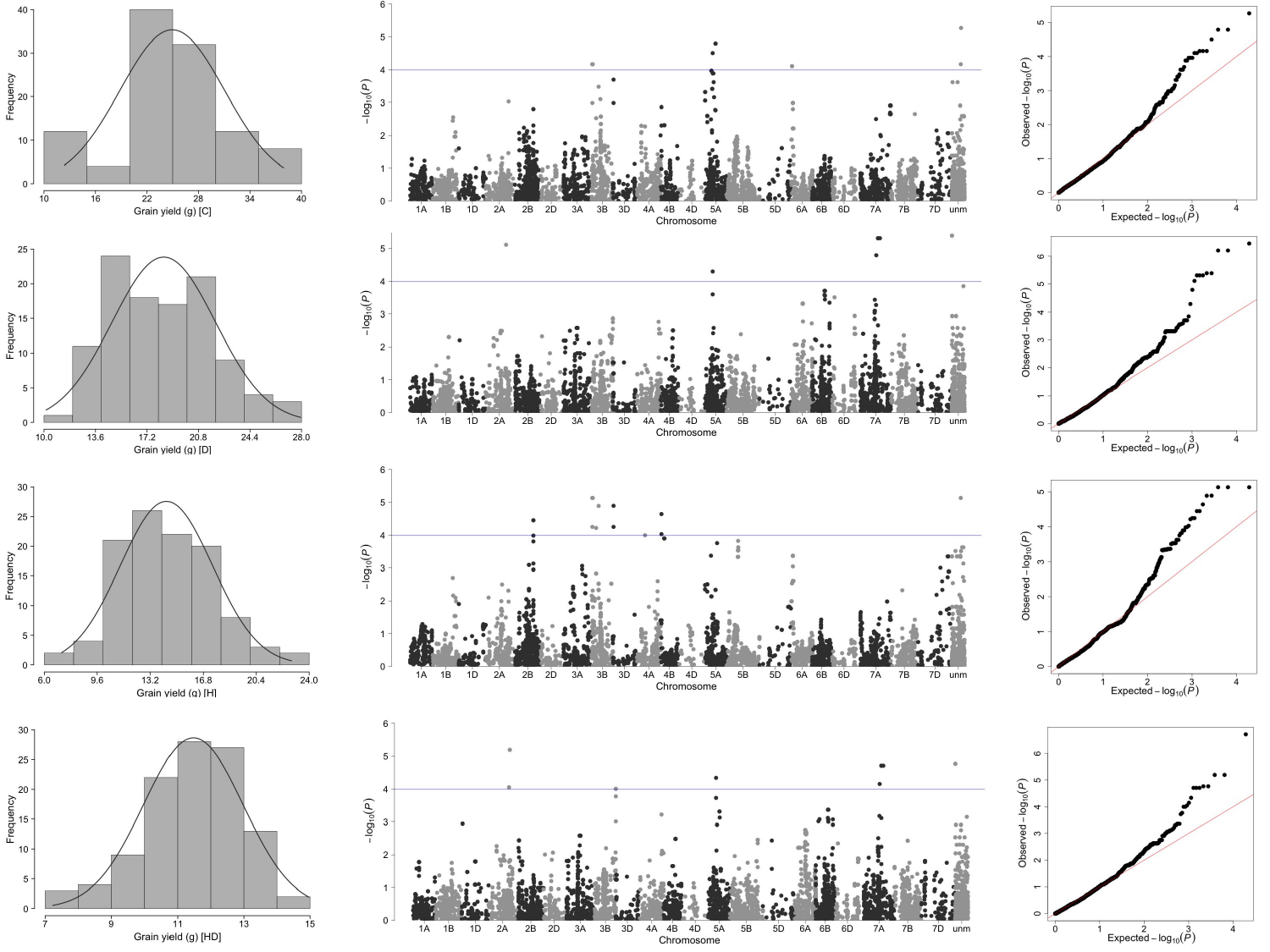

## Slide 8
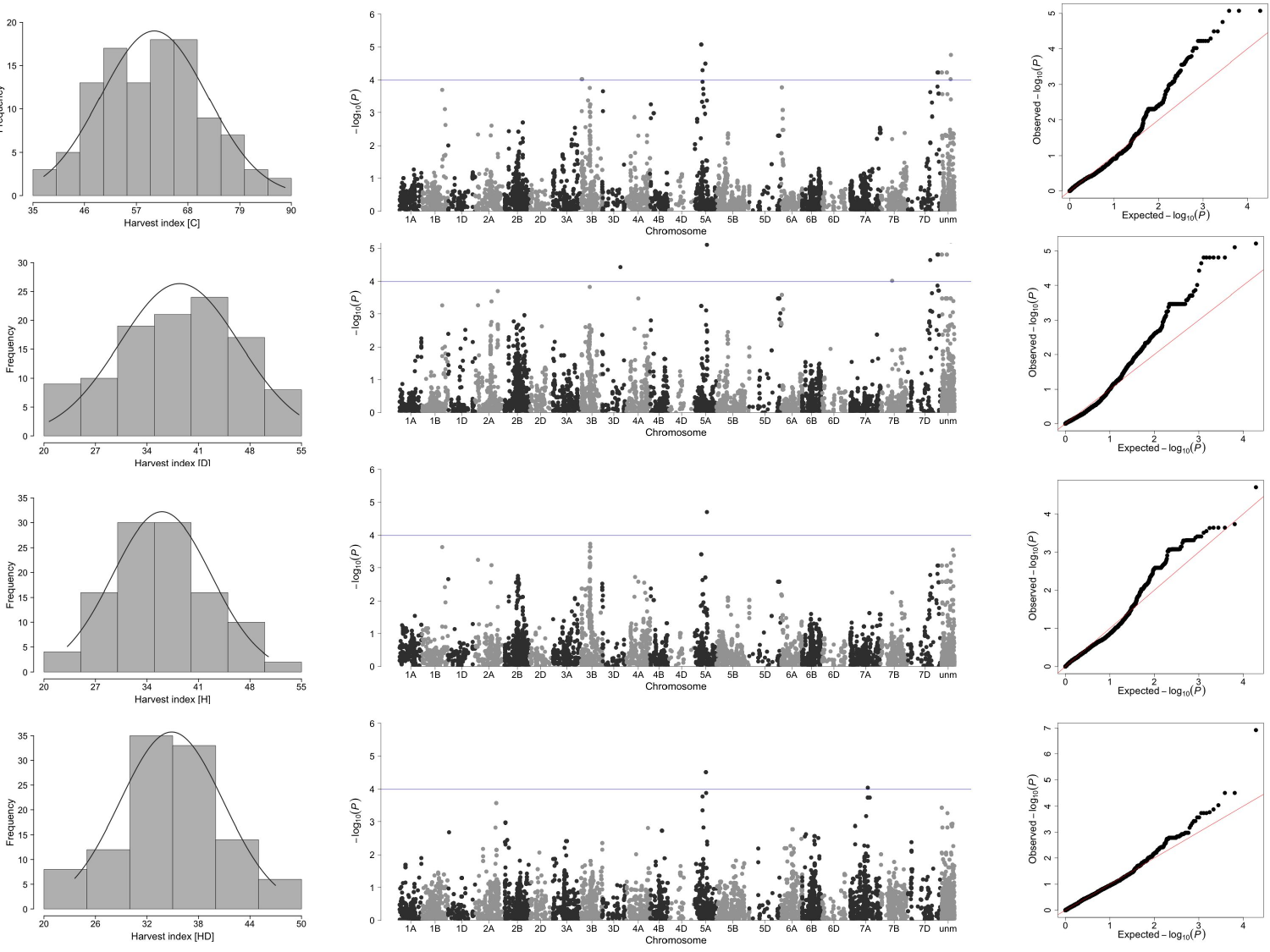

## Slide 9
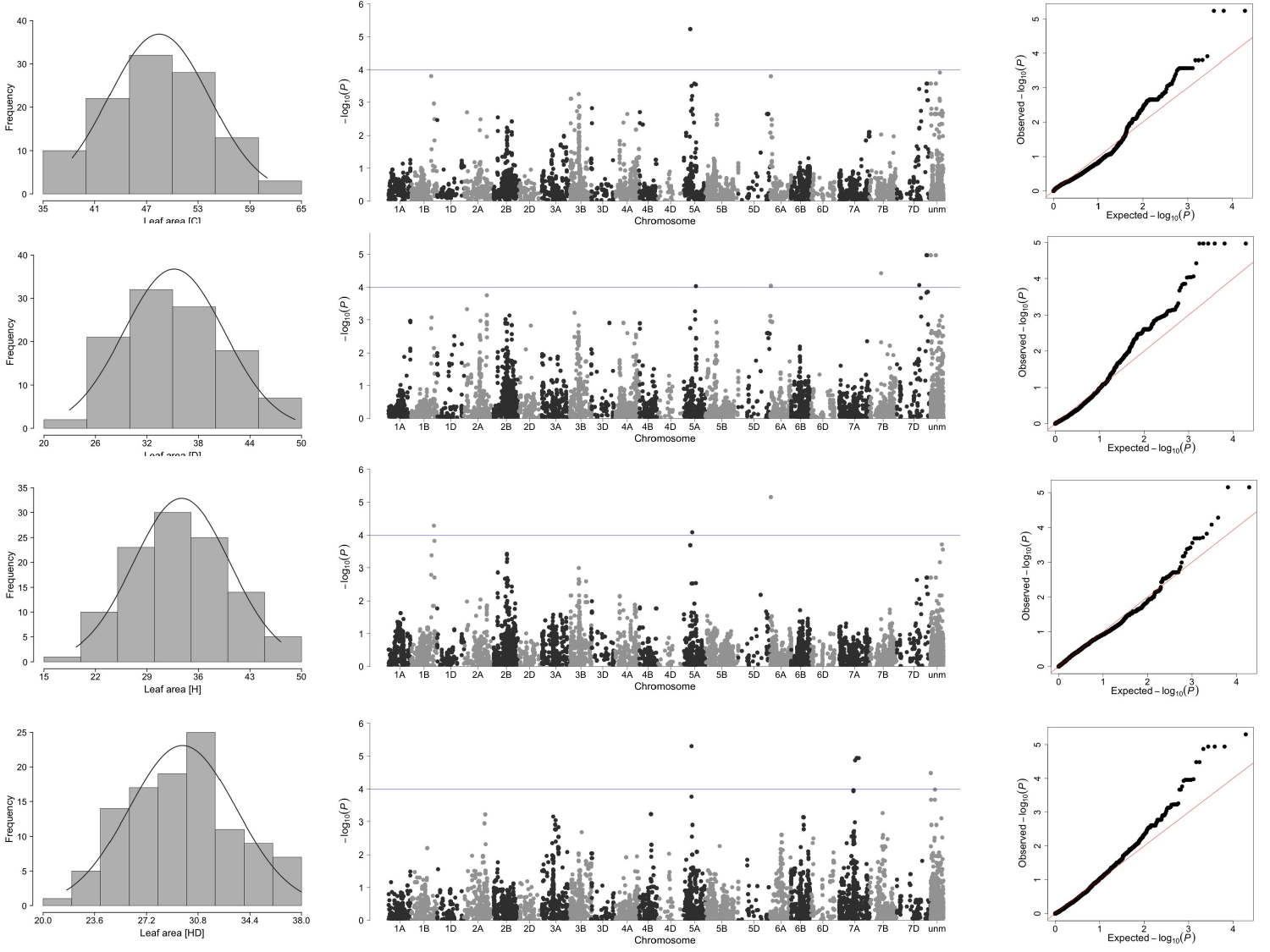

## Slide 10
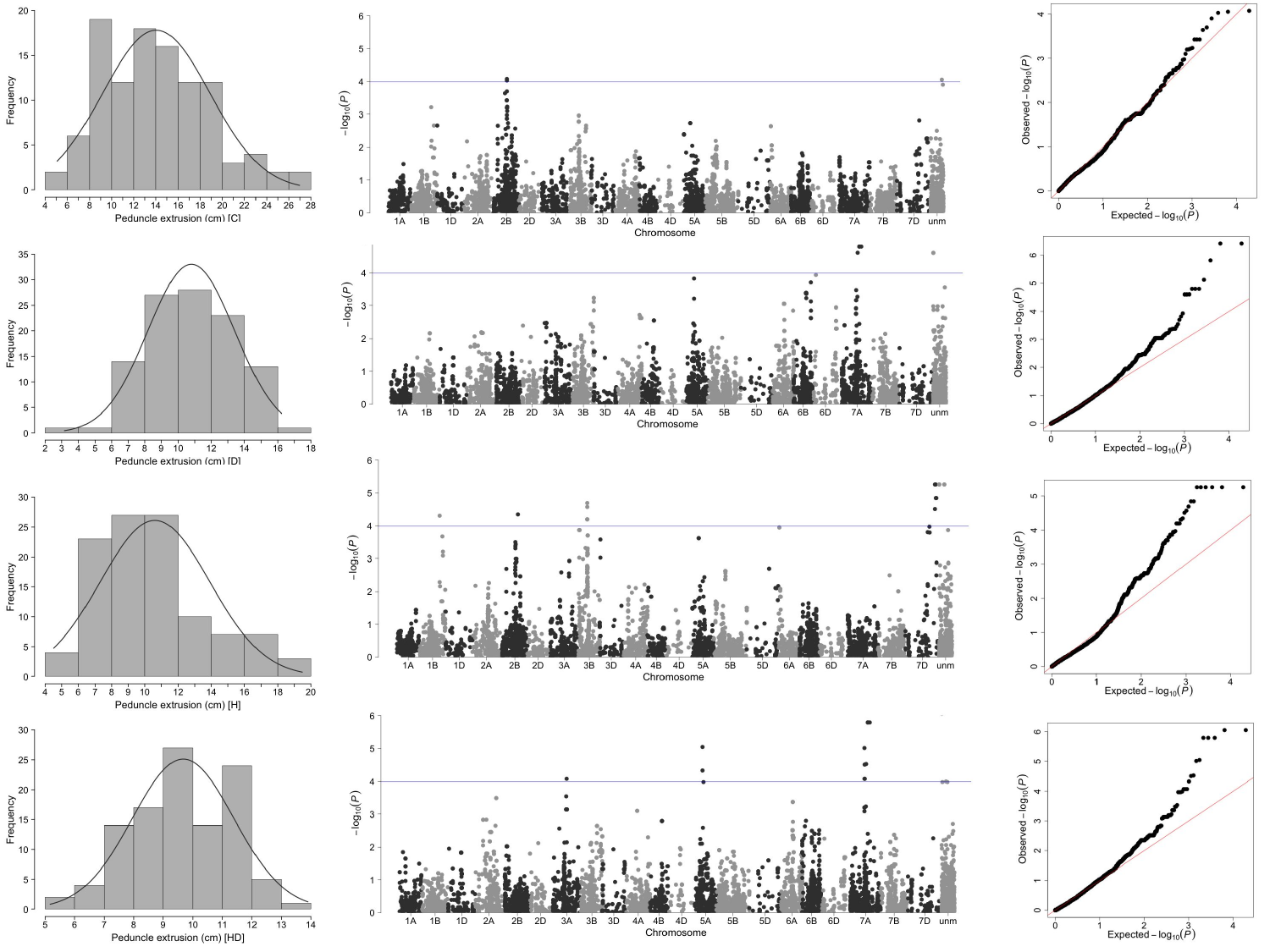

## Slide 11
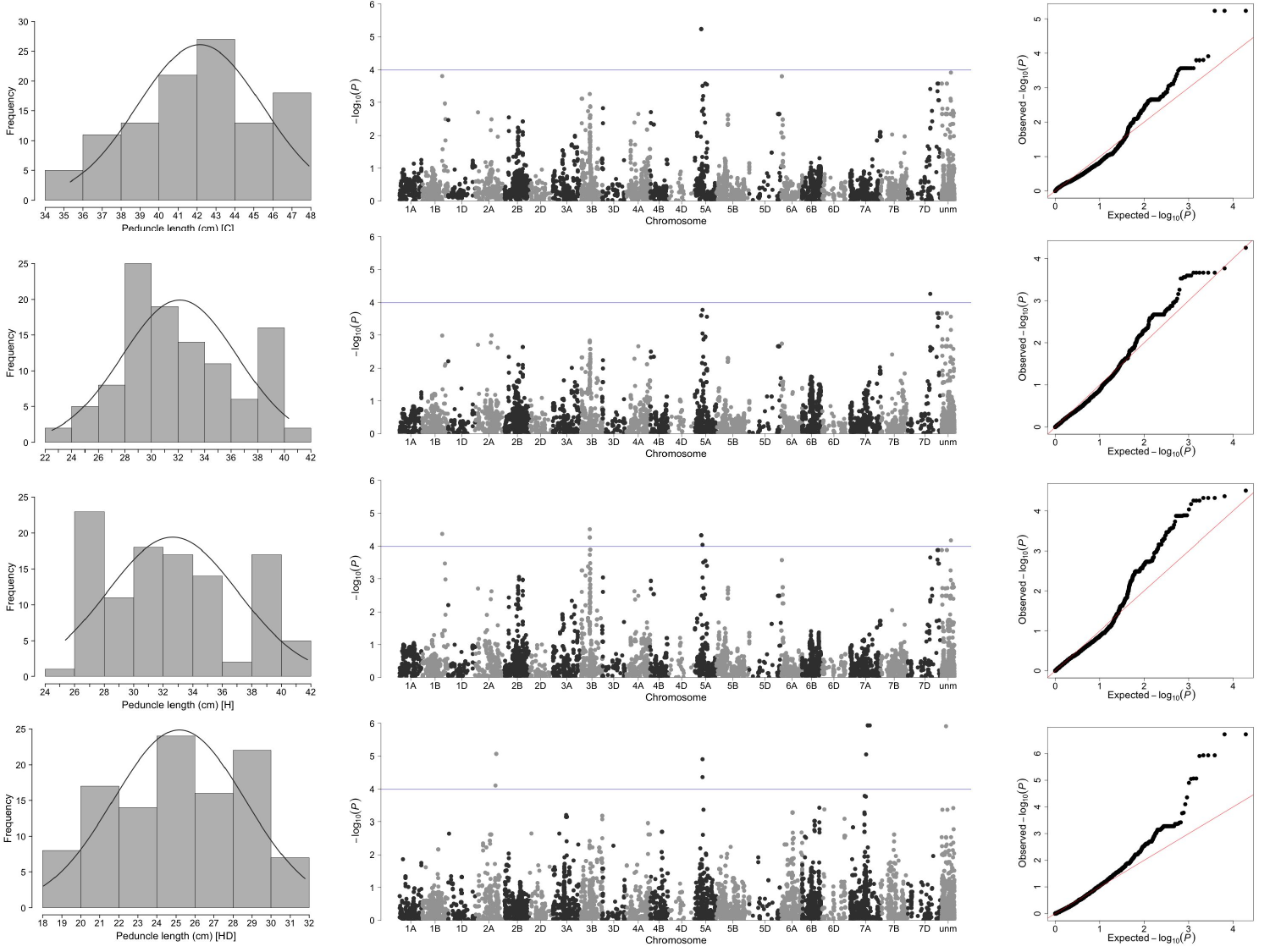

## Slide 12
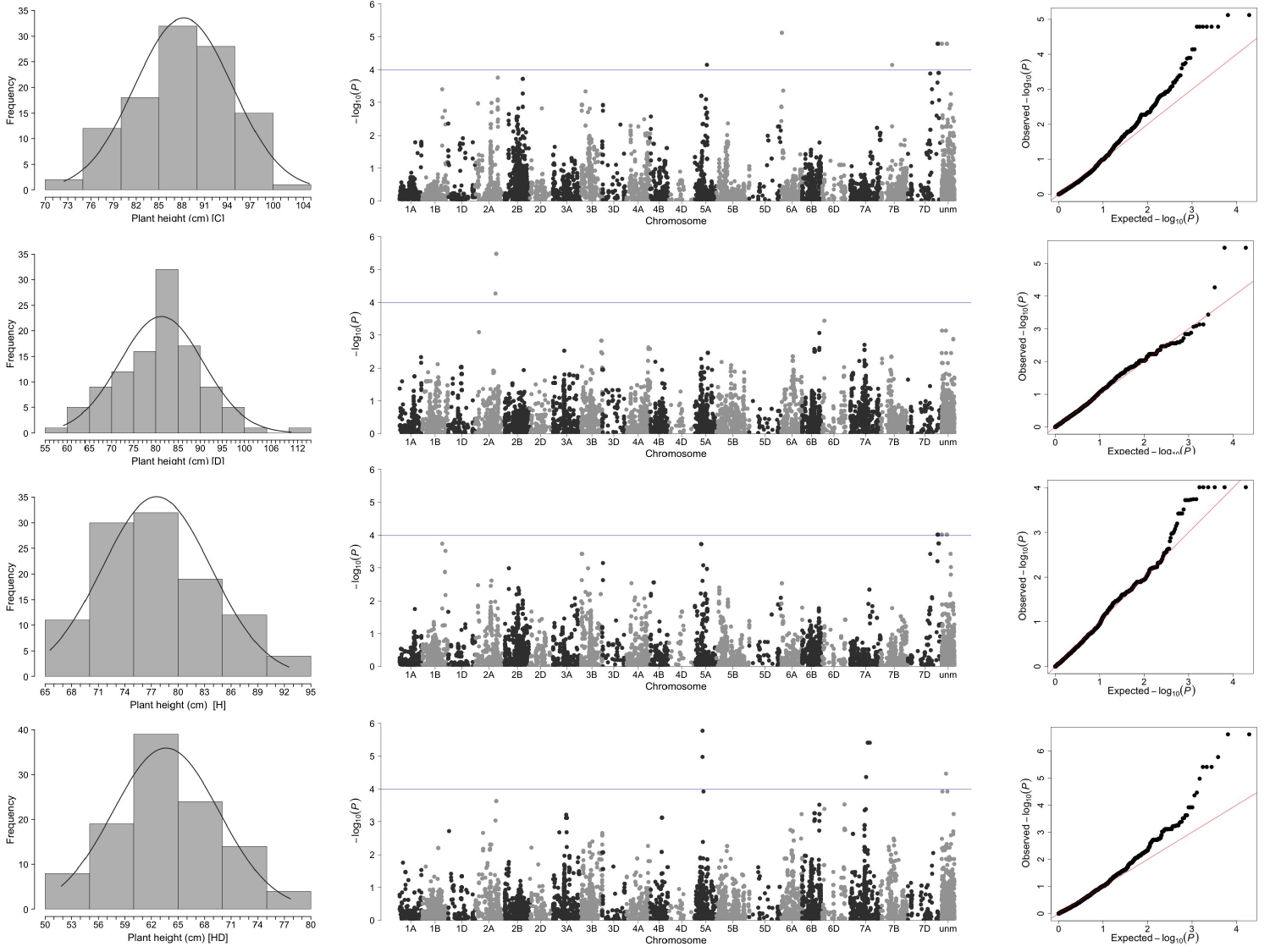

## Slide 13
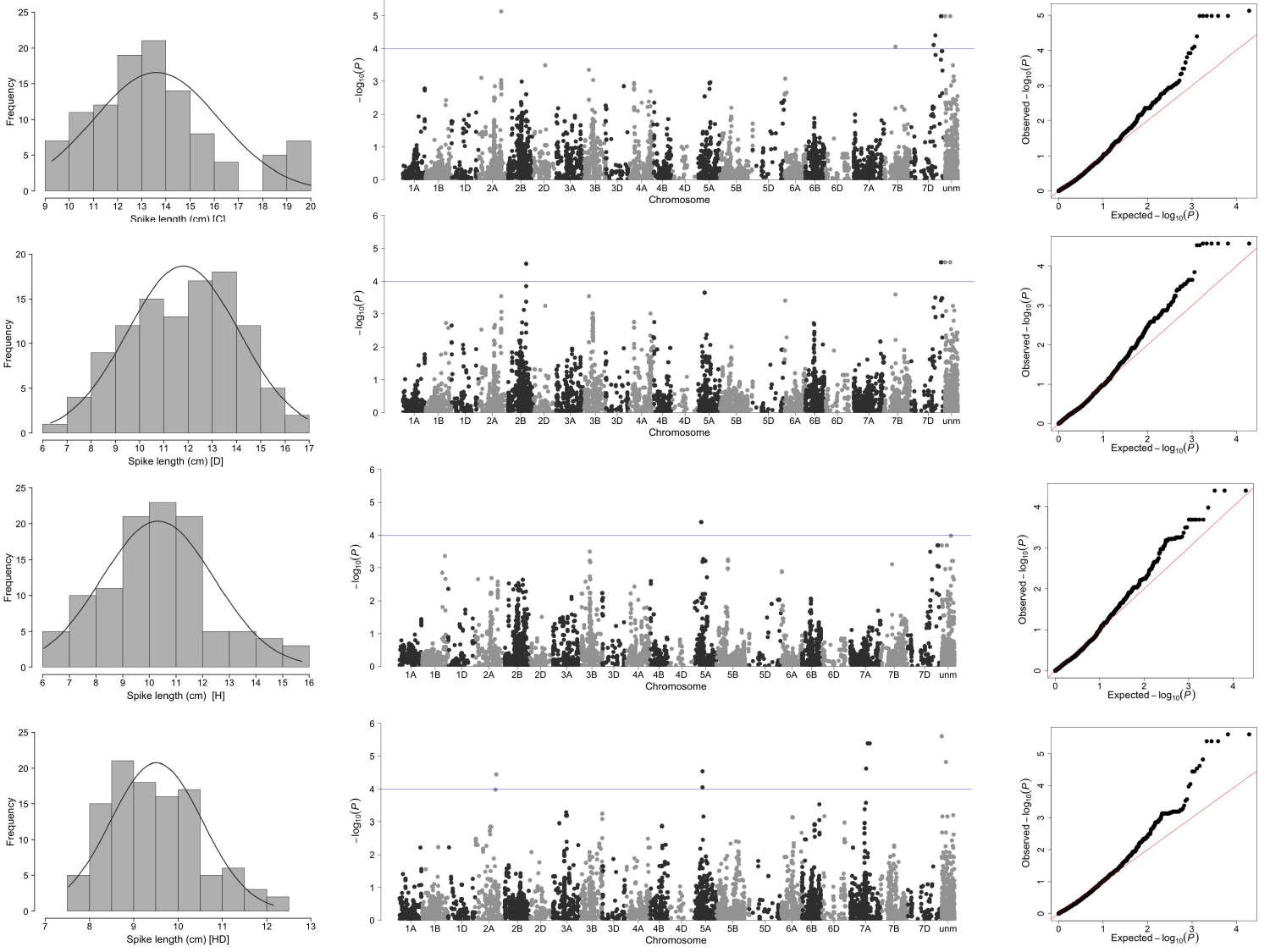

## Slide 14
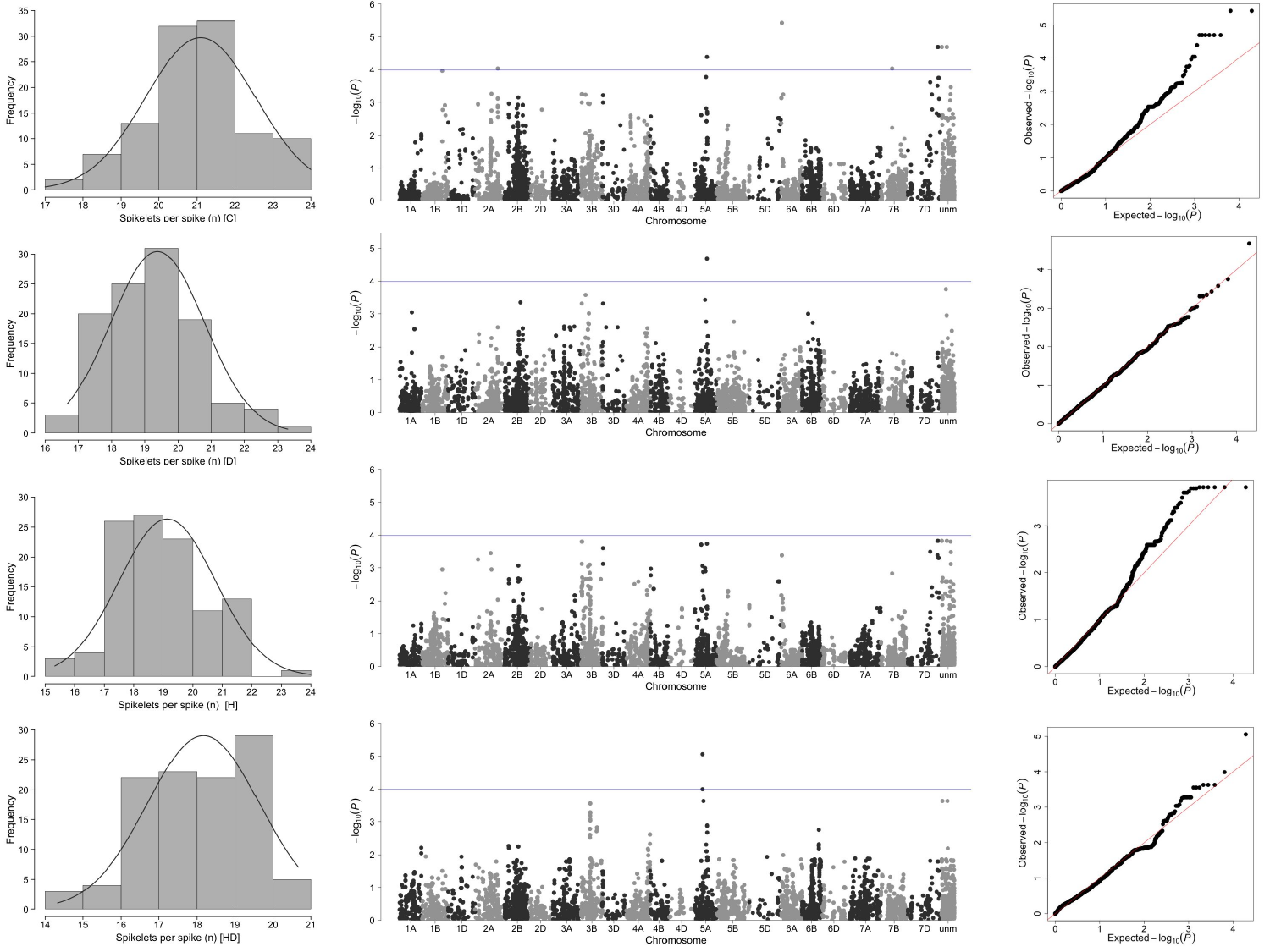

## Slide 15
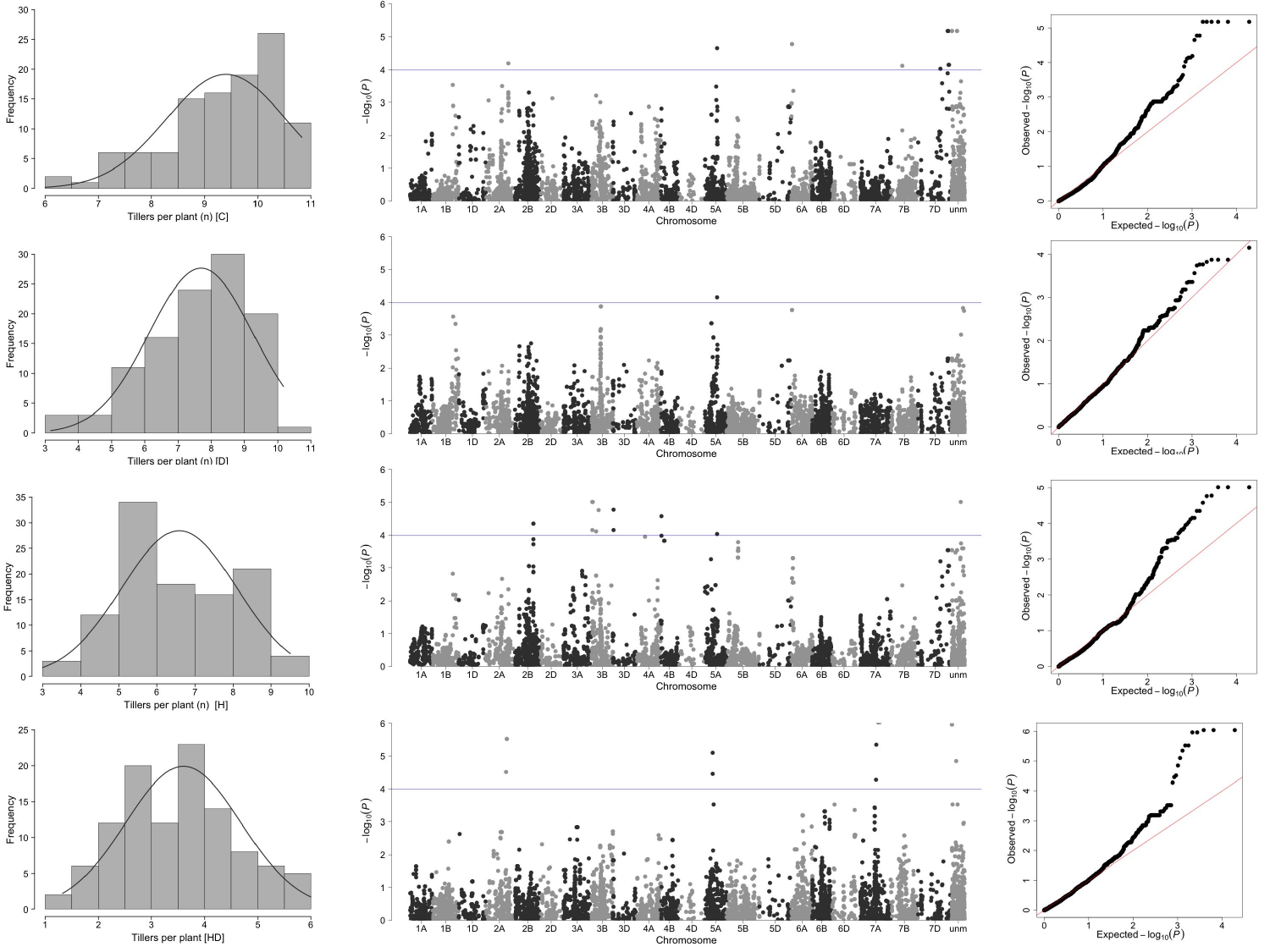

Supplement: S3 File — (PPTX) [file pone.0199121.s014.pptx]
