# Supplementary material for: Genome-wide association mapping in bread wheat subjected to independent and combined high temperature and drought stress
Source: PLoS One. 2018 Jun 27;13(6):e0199121. doi: 10.1371/journal.pone.0199121 (PMC6021117; doi:10.1371/journal.pone.0199121)
Supplement: S1 File — (PDF) [file pone.0199121.s012.pdf]

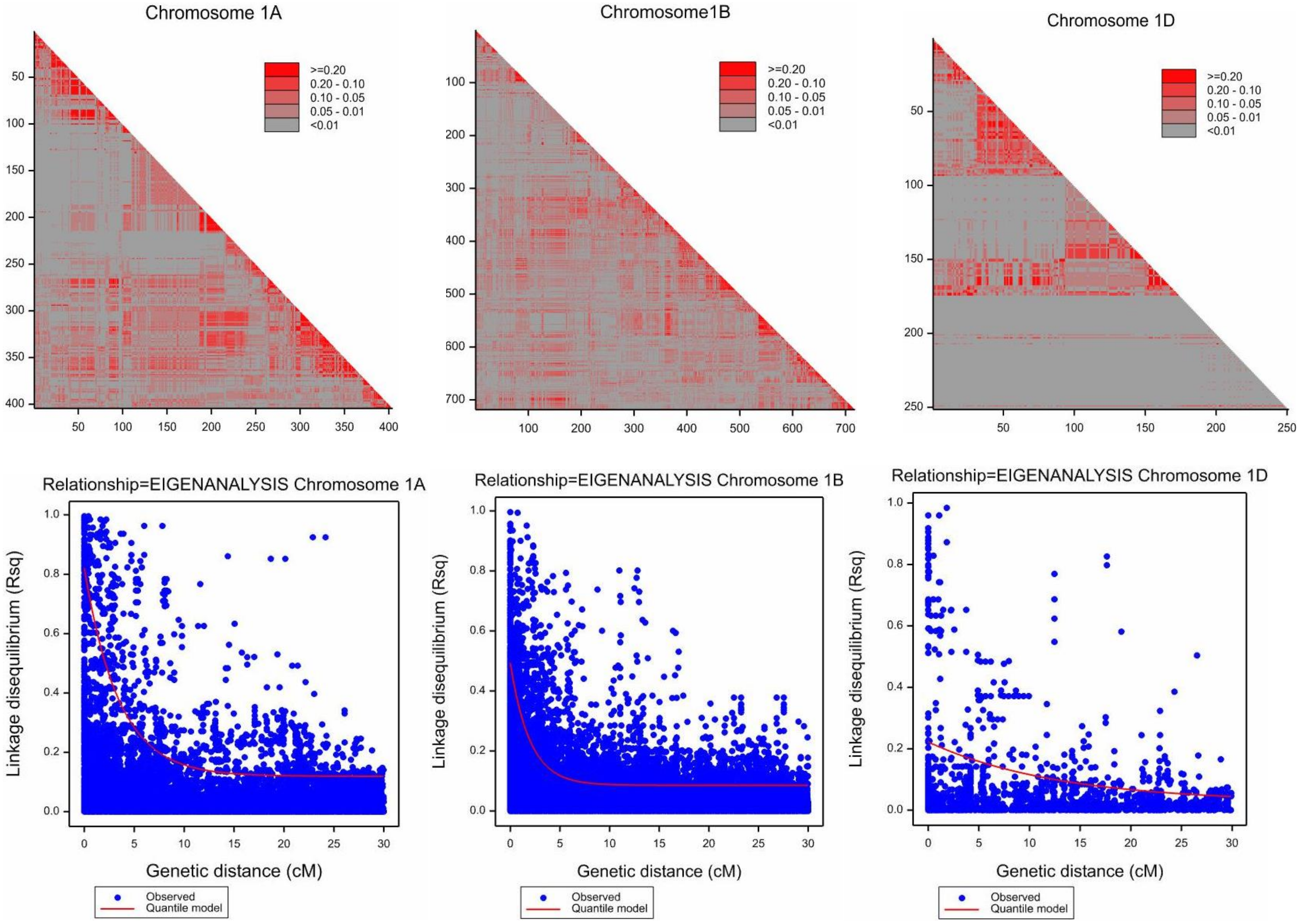

Chromosome 1A
Chromosome1B
Chromosome 1D
>=0.20
0.20 - 0.10
0.10 - 0.05
0.05 - 0.01
<0.01
Relationship=EIGENANALYSIS Chromosome 1A
Relationship=EIGENANALYSIS Chromosome 1B
Relationship=EIGENANALYSIS Chromosome 1D
Linkage disequilibrium (Rsq)
Genetic distance (cM)
Observed
Quantile model

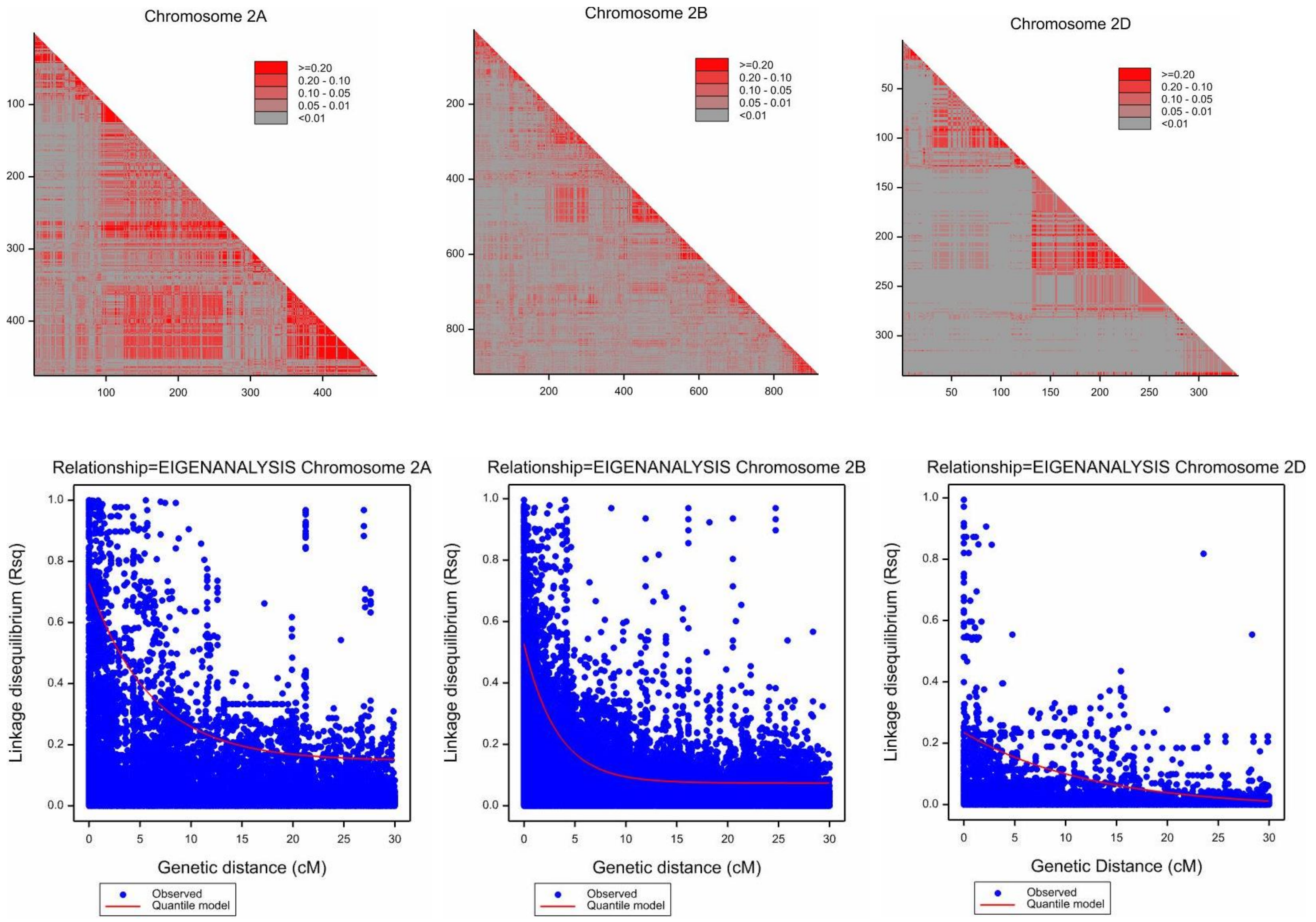

Chromosome 2A
Chromosome 2B
Chromosome 2D
>=0.20
0.20 - 0.10
0.10 - 0.05
0.05 - 0.01
<0.01
Relationship=EIGENANALYSIS Chromosome 2A
Relationship=EIGENANALYSIS Chromosome 2B
Relationship=EIGENANALYSIS Chromosome 2D
Linkage disequilibrium (Rsq)
Genetic distance (cM)
Genetic Distance (cM)
Observed
Quantile model

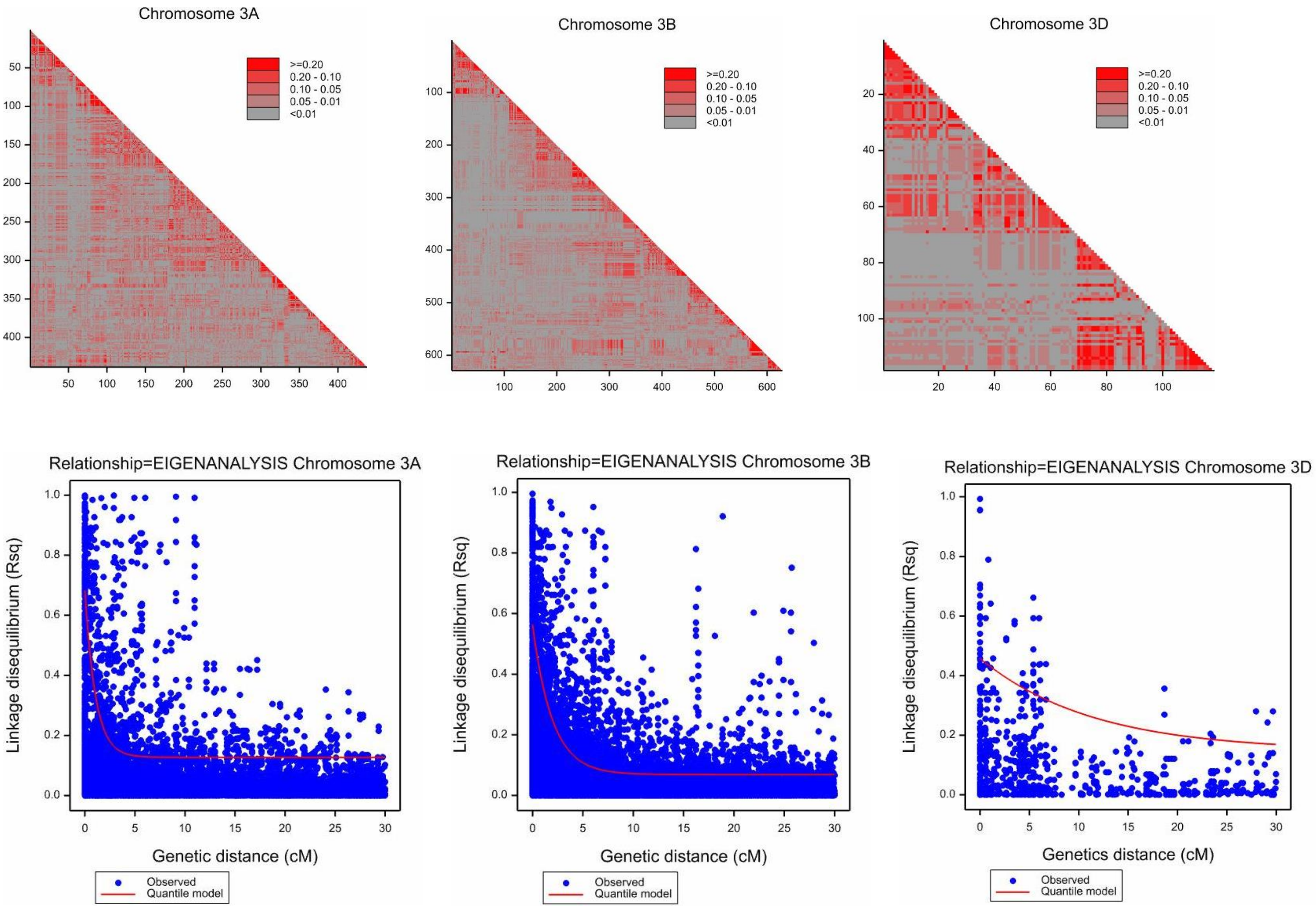
Chromosome 3A
Chromosome 3B
Chromosome 3D
>=0.20
0.20 - 0.10
0.10 - 0.05
0.05 - 0.01
<0.01
Relationship=EIGENANALYSIS Chromosome 3A
Relationship=EIGENANALYSIS Chromosome 3B
Relationship=EIGENANALYSIS Chromosome 3D
Linkage disequilibrium (Rsq)
Genetic distance (cM)
Genetics distance (cM)
Observed
Quantile model

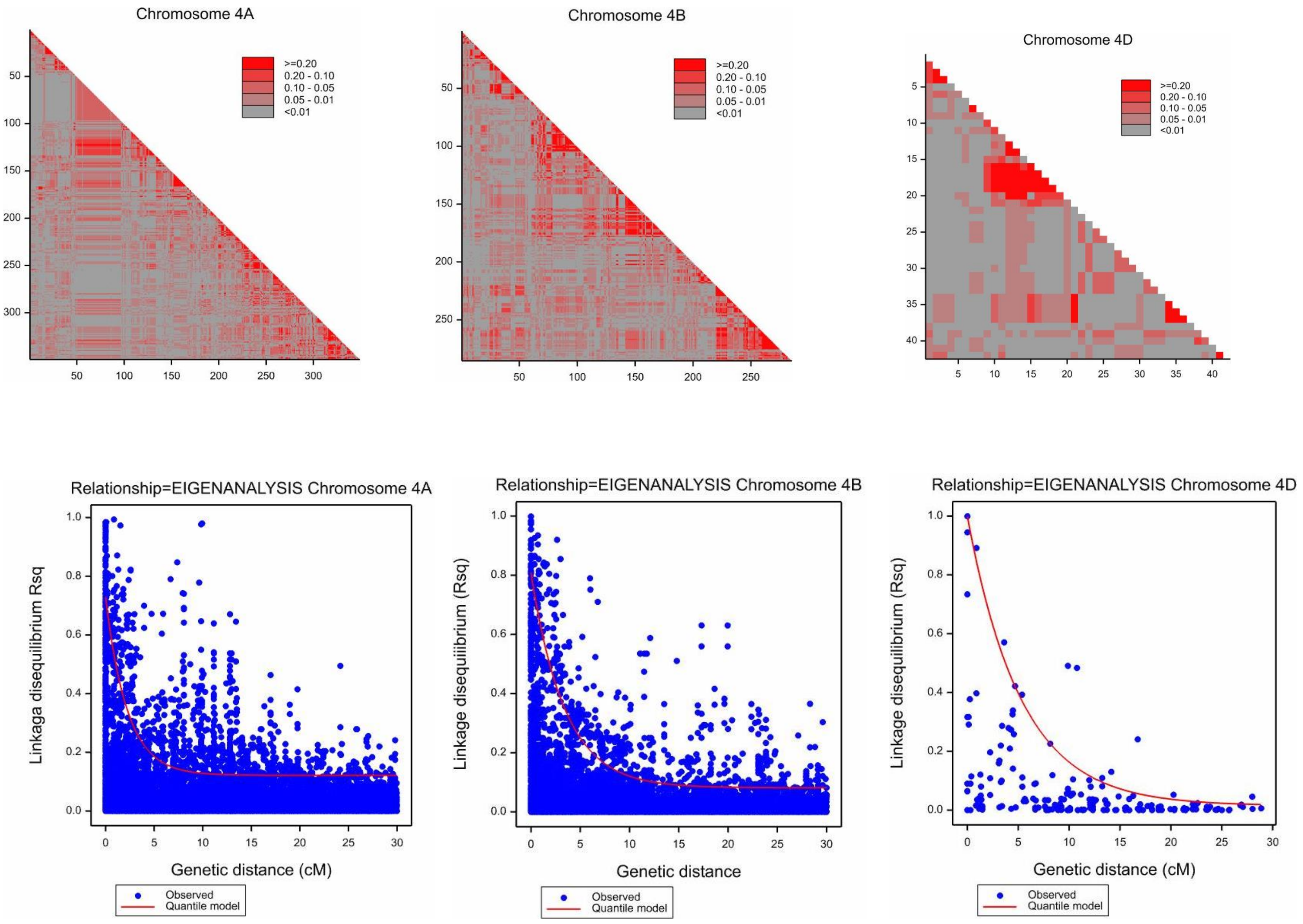
Chromosome 4A
>=0.20
0.20 - 0.10
0.10 - 0.05
0.05 - 0.01
<0.01
Chromosome 4B
Chromosome 4D
Relationship=EIGENANALYSIS Chromosome 4A
Linkaga disequilibrium Rsq
Genetic distance (cM)
Observed
Quantile model
Relationship=EIGENANALYSIS Chromosome 4B
Linkage disequilibrium (Rsq)
Genetic distance
Relationship=EIGENANALYSIS Chromosome 4D
Linkage disequilibrium (Rsq)
Genetic distance (cM)

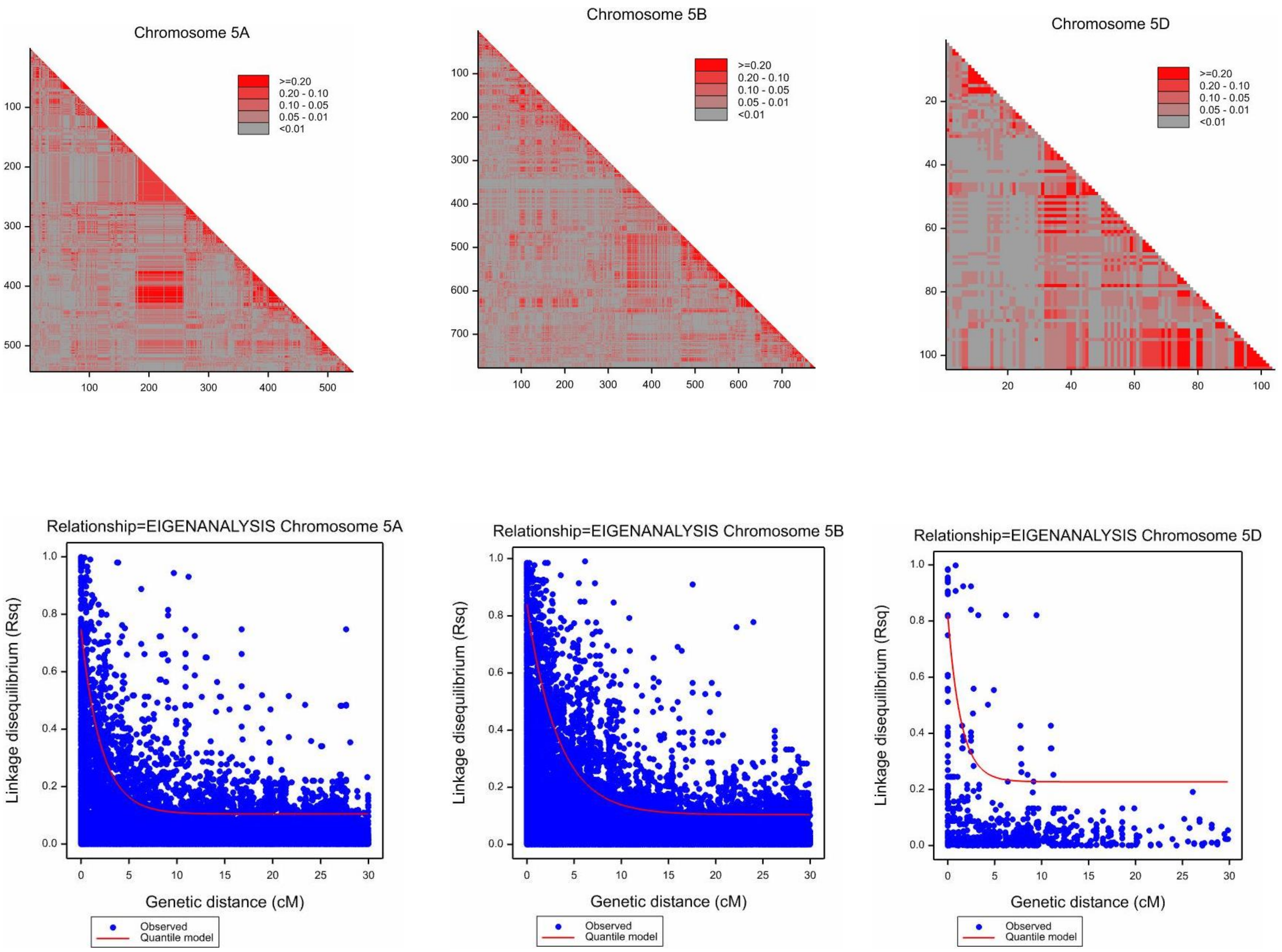

Chromosome 5A
>=0.20
0.20 - 0.10
0.10 - 0.05
0.05 - 0.01
<0.01
Chromosome 5B
>=0.20
0.20 - 0.10
0.10 - 0.05
0.05 - 0.01
<0.01
Chromosome 5D
>=0.20
0.20 - 0.10
0.10 - 0.05
0.05 - 0.01
<0.01
Relationship=EIGENANALYSIS Chromosome 5A
Linkage disequilibrium (Rsq)
Genetic distance (cM)
Observed
Quantile model
Relationship=EIGENANALYSIS Chromosome 5B
Linkage disequilibrium (Rsq)
Genetic distance (cM)
Observed
Quantile model
Relationship=EIGENANALYSIS Chromosome 5D
Linkage disequilibrium (Rsq)
Genetic distance (cM)
Observed
Quantile model

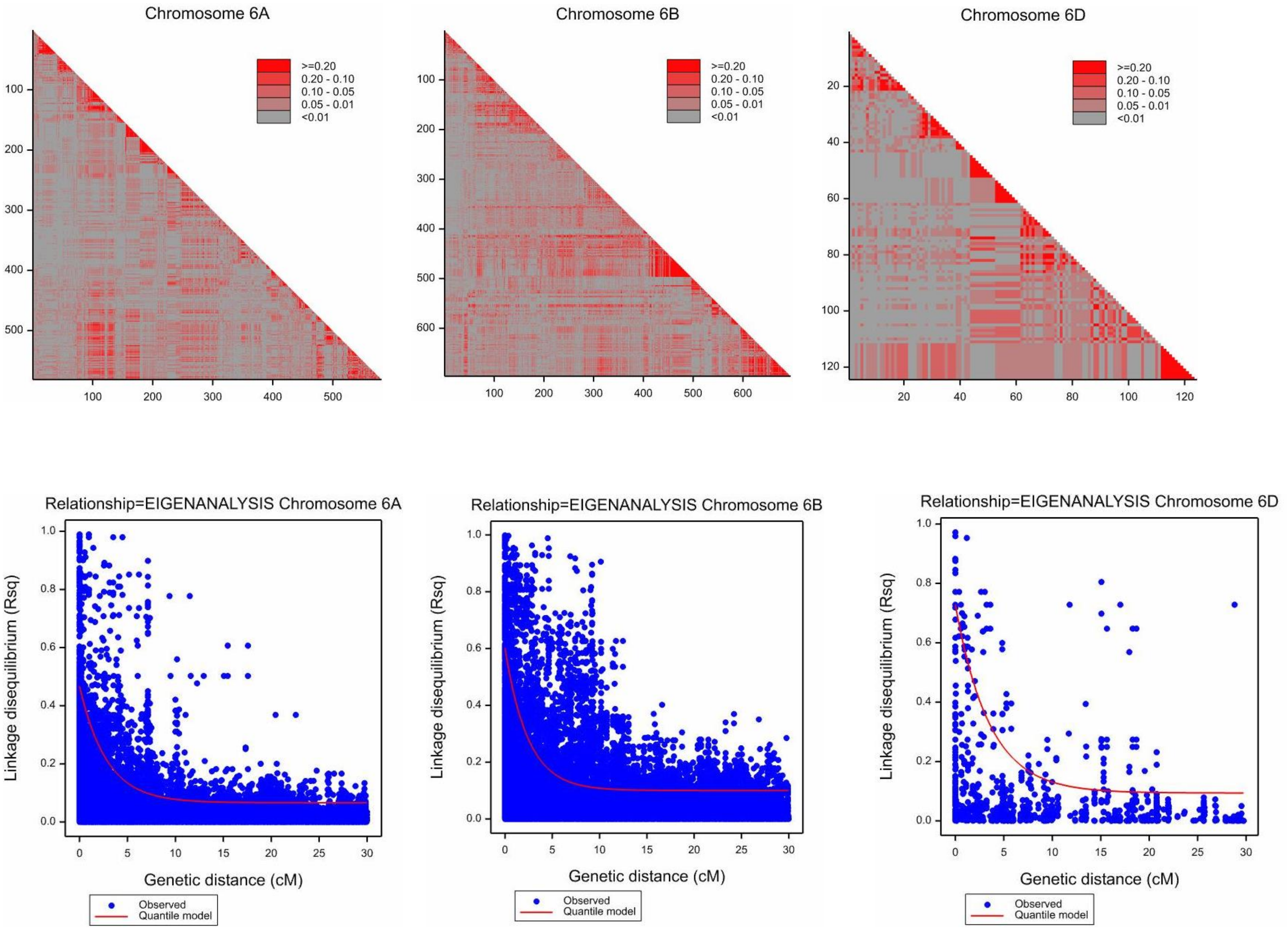

Chromosome 6A
Chromosome 6B
Chromosome 6D
>=0.20
0.20 - 0.10
0.10 - 0.05
0.05 - 0.01
<0.01
Relationship=EIGENANALYSIS Chromosome 6A
Relationship=EIGENANALYSIS Chromosome 6B
Relationship=EIGENANALYSIS Chromosome 6D
Linkage disequilibrium (Rsq)
Genetic distance (cM)
Observed
Quantile model

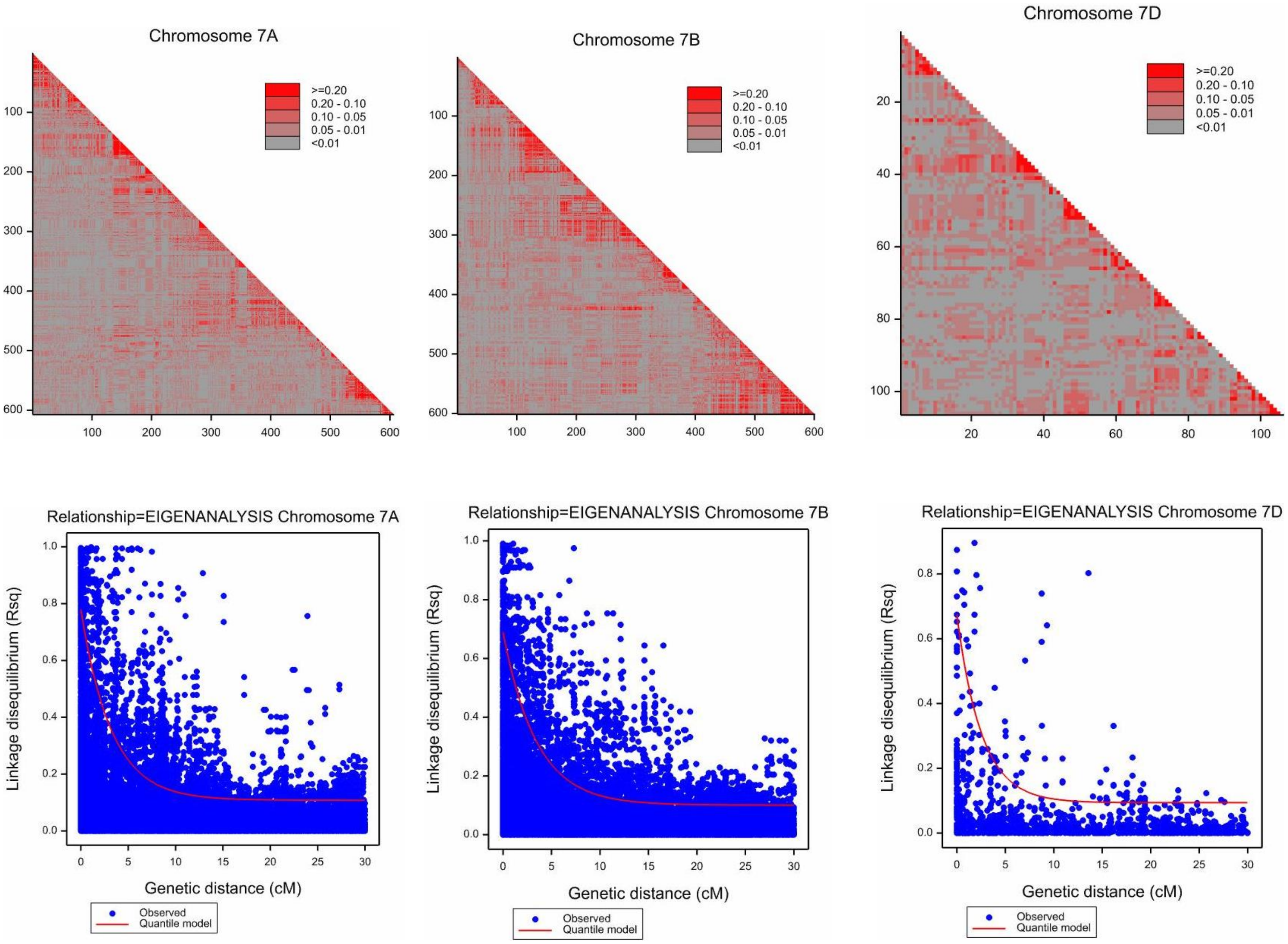
Chromosome 7A
Chromosome 7B
Chromosome 7D
>=0.20
0.20 - 0.10
0.10 - 0.05
0.05 - 0.01
<0.01
Relationship=EIGENANALYSIS Chromosome 7A
Relationship=EIGENANALYSIS Chromosome 7B
Relationship=EIGENANALYSIS Chromosome 7D
Linkage disequilibrium (Rsq)
Genetic distance (cM)
Observed
Quantile model
