# Supplementary material for: Genome-wide association mapping in bread wheat subjected to independent and combined high temperature and drought stress
Source: PLoS One. 2018 Jun 27;13(6):e0199121. doi: 10.1371/journal.pone.0199121 (PMC6021117; doi:10.1371/journal.pone.0199121)
Supplement: S2 File — (PDF) [file pone.0199121.s013.pdf]

1B

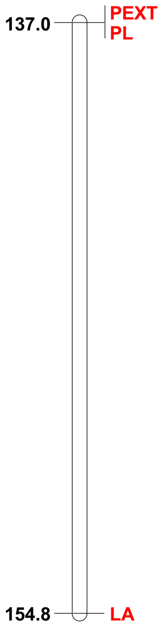

●	Control
●	Drought
●	Heat
●	Combination

AWL: Awn length, Biomass: Above ground plant dry weight, DTA: Days to anthesis, DTH: Days to heading, DTM: Days to maturity, GPS: Grains per spike, GY: Grain yield, HI: Harvest index, LA: Leaf area, PL: Peduncle length, Pext: Peduncle extrusion, PH: Plant height, SLP: Spikelets per spike, SL: Spike length, Till: Tillers per plant,

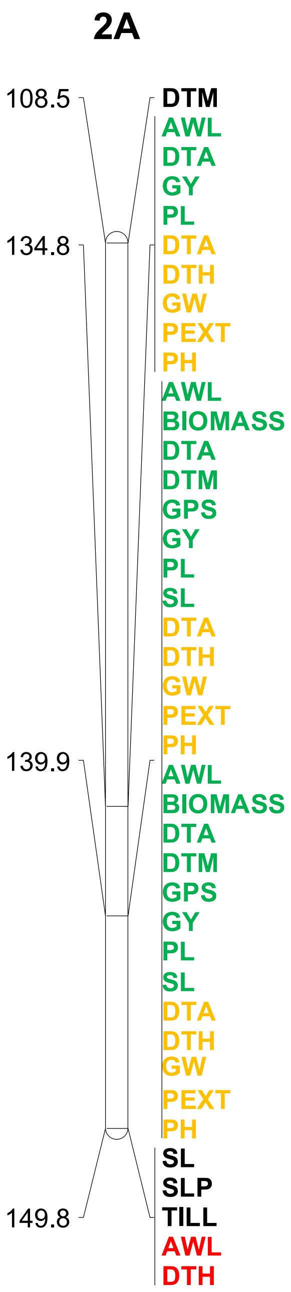

●	Control
●	Drought
●	Heat
●	Combination

AWL: Awn length, Biomass: Above ground plant dry weight, DTA: Days to anthesis, DTH: Days to heading, DTM: Days to maturity, GPS: Grains per spike, GY: Grain yield, HI: Harvest index, LA: Leaf area, PL: Peduncle length, Pext: Peduncle extrusion, PH: Plant height, SLP: Spikelets per spike, SL: Spike length, Till: Tillers per plant,

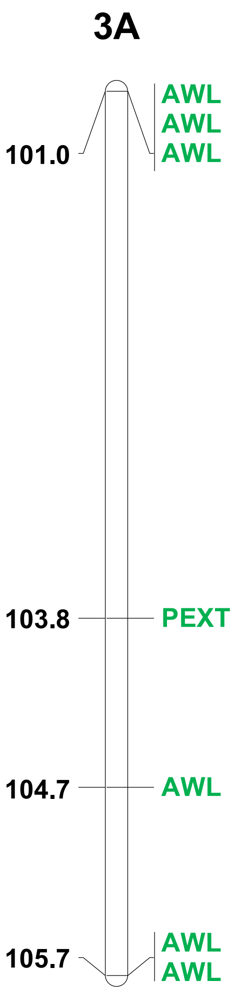

●	Control
●	Drought
●	Heat
●	Combination

AWL: Awn length, Biomass: Above ground plant dry weight, DTA: Days to anthesis, DTH: Days to heading, DTM: Days to maturity, GPS: Grains per spike, GY: Grain yield, HI: Harvest index, LA: Leaf area, PL: Peduncle length, Pext: Peduncle extrusion, PH: Plant height, SLP: Spikelets per spike, SL: Spike length, Till: Tillers per plant,

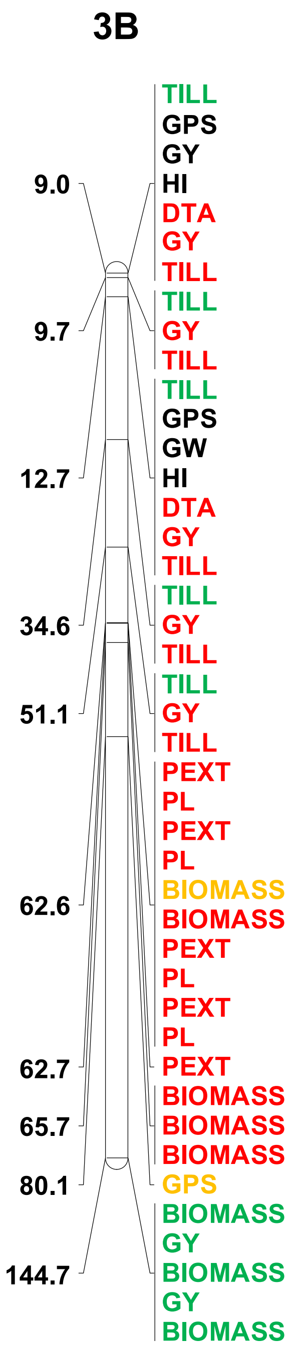

	Control
	Drought
	Heat
	Combination

AWL: Awn length, Biomass: Above ground plant dry weight, DTA: Days to anthesis, DTH: Days to heading, DTM: Days to maturity, GPS: Grains per spike, GY: Grain yield, HI: Harvest index, LA: Leaf area, PL: Peduncle length, Pext: Peduncle extrusion, PH: Plant height, SLP: Spikelets per spike, SL: Spike length, Till: Tillers per plant,

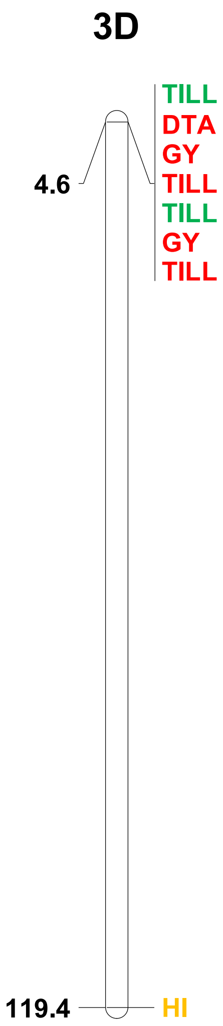

●	Control
●	Drought
●	Heat
●	Combination

AWL: Awn length, Biomass: Above ground plant dry weight, DTA: Days to anthesis, DTH: Days to heading, DTM: Days to maturity, GPS: Grains per spike, GY: Grain yield, HI: Harvest index, LA: Leaf area, PL: Peduncle length, Pext: Peduncle extrusion, PH: Plant height, SLP: Spikelets per spike, SL: Spike length, Till: Tillers per plant,

5A

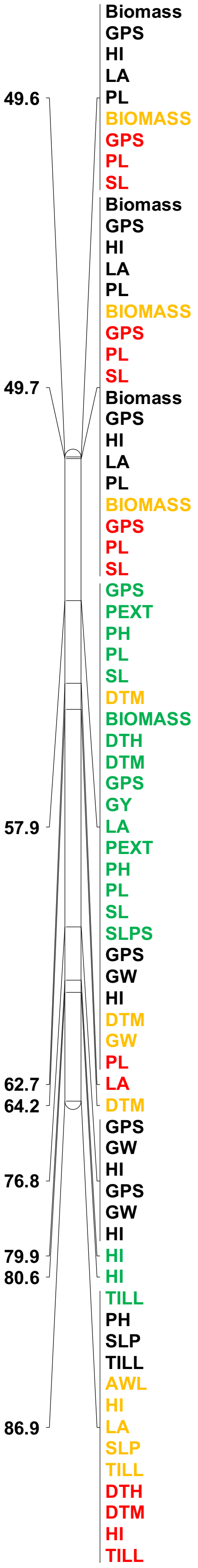
49.6
49.7
57.9
62.7
64.2
76.8
79.9
80.6
86.9
Biomass
GPS
HI
LA
PL
BIOMASS
GPS
PL
SL
Biomass
GPS
HI
LA
PL
BIOMASS
GPS
PL
SL
Biomass
GPS
HI
LA
PL
BIOMASS
GPS
PL
SL
GPS
PEXT
PH
PL
SL
DTM
BIOMASS
DTH
DTM
GPS
GY
LA
PEXT
PH
PL
SL
SLPS
GPS
GW
HI
DTM
GW
PL
LA
DTM
GPS
GW
HI
GPS
GW
HI
HI
HI
TILL
PH
SLP
TILL
AWL
HI
LA
SLP
TILL
DTH
DTM
HI
TILL

6A

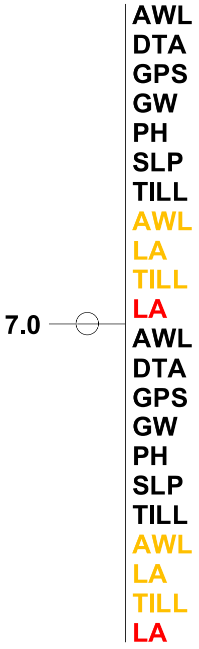

	Control
	Drought
	Heat
	Combination

AWL: Awn length, Biomass: Above ground plant dry weight, DTA: Days to anthesis, DTH: Days to heading, DTM: Days to maturity, GPS: Grains per spike, GY: Grain yield, HI: Harvest index, LA: Leaf area, PL: Peduncle length, Pext: Peduncle extrusion, PH: Plant height, SLP: Spikelets per spike, SL: Spike length, Till: Tillers per plant,

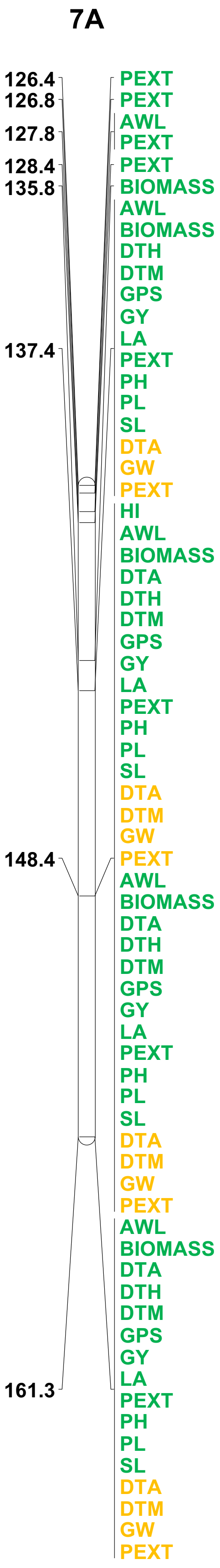
7A
126.4
126.8
127.8
128.4
135.8
137.4
148.4
161.3
PEXT
PEXT
AWL
PEXT
PEXT
BIOMASS
AWL
BIOMASS
DTH
DTM
GPS
GY
LA
PEXT
PH
PL
SL
DTA
GW
PEXT
HI
AWL
BIOMASS
DTA
DTH
DTM
GPS
GY
LA
PEXT
PH
PL
SL
DTA
DTM
GW
PEXT
AWL
BIOMASS
DTA
DTH
DTM
GPS
GY
LA
PEXT
PH
PL
SL
DTA
DTM
GW
PEXT
AWL
BIOMASS
DTA
DTH
DTM
GPS
GY
LA
PEXT
PH
PL
SL
DTA
DTM
GW
PEXT

7B

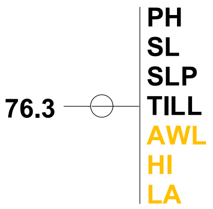

●	Control
●	Drought
●	Heat
●	Combination

AWL: Awn length, Biomass: Above ground plant dry weight, DTA: Days to anthesis, DTH: Days to heading, DTM: Days to maturity, GPS: Grains per spike, GY: Grain yield, HI: Harvest index, LA: Leaf area, PL: Peduncle length, Pext: Peduncle extrusion, PH: Plant height, SLP: Spikelets per spike, SL: Spike length, Till: Tillers per plant,

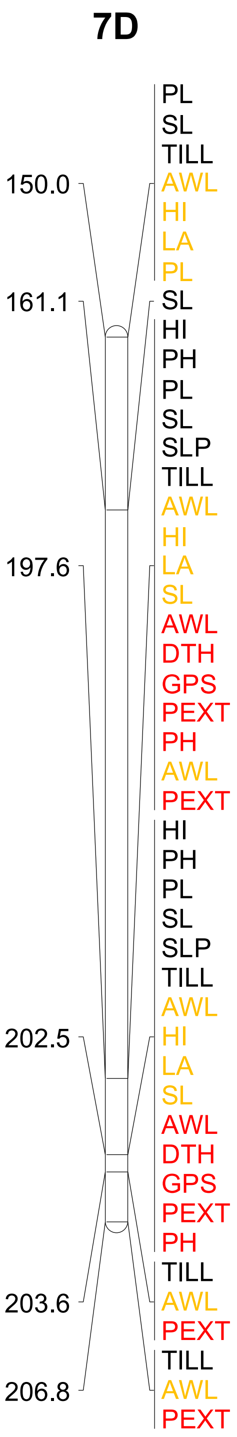

●	Control
●	Drought
●	Heat
●	Combination

AWL: Awn length, Biomass: Above ground plant dry weight, DTA: Days to anthesis, DTH: Days to heading, DTM: Days to maturity, GPS: Grains per spike, GY: Grain yield, HI: Harvest index, LA: Leaf area, PL: Peduncle length, Pext: Peduncle extrusion, PH: Plant height, SLP: Spikelets per spike, SL: Spike length, Till: Tillers per plant,
